# The distribution and abundance of archaeal tetraether lipids in U.S. Great Basin hot springs

**DOI:** 10.3389/fmicb.2013.00247

**Published:** 2013-08-28

**Authors:** Julienne J. Paraiso, Amanda J. Williams, Qiuyuan Huang, Yuli Wei, Paul Dijkstra, Bruce A. Hungate, Hailiang Dong, Brian P. Hedlund, Chuanlun L. Zhang

**Affiliations:** ^1^State Key Laboratory of Marine Geology, Tongji UniversityShanghai, China; ^2^School of Life Sciences, University of Nevada, Las VegasLas Vegas, NV, USA; ^3^Department of Geology, Miami UniversityOxford, OH, USA; ^4^Department of Marine Sciences, University of GeorgiaAthens, GA, USA; ^5^Department of Biological Sciences and Merriam-Powell Center for Environmental Research, Northern Arizona UniversityFlagstaff, AZ, USA

**Keywords:** archaea, iGDGTs, hot springs, Great Basin, lipids

## Abstract

Isoprenoidal glycerol dialkyl glycerol tetraethers (iGDGTs) are core membrane lipids of many archaea that enhance the integrity of cytoplasmic membranes in extreme environments. We examined the iGDGT profiles and corresponding aqueous geochemistry in 40 hot spring sediment and microbial mat samples from the U.S. Great Basin with temperatures ranging from 31 to 95°C and pH ranging from 6.8 to 10.7. The absolute abundance of iGDGTs correlated negatively with pH and positively with temperature. High lipid concentrations, distinct lipid profiles, and a strong relationship between polar and core lipids in hot spring samples suggested *in situ* production of most iGDGTs rather than contamination from local soils. Two-way cluster analysis and non-metric multidimensional scaling (NMS) of polar iGDGTs indicated that the relative abundance of individual lipids was most strongly related to temperature (*r*^2^ = 0.546), with moderate correlations with pH (*r*^2^ = 0.359), nitrite (*r*^2^ = 0.286), oxygen (*r*^2^ = 0.259), and nitrate (*r*^2^ = 0.215). Relative abundance profiles of individual polar iGDGTs indicated potential temperature optima for iGDGT-0 (≤70°C), iGDGT-3 (≥55°C), and iGDGT-4 (≥60°C). These relationships likely reflect both physiological adaptations and community-level population shifts in response to temperature differences, such as a shift from cooler samples with more abundant methanogens to higher-temperature samples with more abundant Crenarchaeota. Crenarchaeol was widely distributed across the temperature gradient, which is consistent with other reports of abundant crenarchaeol in Great Basin hot springs and suggests a wide distribution for thermophilic ammonia-oxidizing archaea (AOA).

## Introduction

The cellular membranes of many archaea have a monolayer architecture comprised of membrane-spanning lipids composed of isoprenoidal chains joined by four ether bonds to two glycerol backbones, known as isoprenoidal GDGTs (iGDGTs; Figure S1) (van de Vossenberg et al., 1998; Schouten et al., 2000). Archaea that produce iGDGTs include thermophilic and hyperthermophilic Thermoprotei (Crenarchaeota), Thaumarchaeota, and at least eight orders of Euryarchaeota including methanogens, thermoacidophiles, and other thermophilic Euryarchaeota (reviewed in Schouten et al., 2013; Pearson and Ingalls, 2013). However, since many major lineages of archaea have not yet been grown axenically (Elkins et al., 2008; Nunoura et al., 2011; Kozubal et al., 2013; Rinke et al., 2013), the known diversity of iGDGT-producing archaea is far from complete.

Microbial ecologists first considered the structure of archaeal tetraether membrane lipids to be an adaptation to life in extreme environments because the covalent linkage between hydrophobic moieties resists thermal and chemical denaturation (Gliozzi et al., 1983). Although some iGDGTs are synthesized by non-extremophilic archaea, particularly crenarchaeol-producing Thaumarchaeota (Sinninghe Damsté et al., 2002; Könneke et al., 2005), the importance of membrane-spanning lipids to life at high temperature and low pH has been supported by a large body of research. Tetraether lipids reduce proton permeability in membrane liposomes (Elferink et al., 1994), and a variety of archaea increase the ratio of tetraether lipids to diether lipids in response to increases in growth temperature or decreases in pH (Sprott et al., 1991; Macalady et al., 2004; Uda et al., 2004; Lai et al., 2008). In addition, several pure culture studies, including Crenarchaeota, Euryarchaeota, and Thaumarchaeota, have shown that archaea increase the number of alkyl group cyclopentane rings, ranging from 0 to 8 per iGDGT, in response to growth temperature increase or pH decrease (Gliozzi et al., 1983; reviewed in De Rosa et al., 1980; Chong, 2010). Molecular modeling studies have shown that increased cyclization stabilizes membranes by tightening membrane packing in the hydrophobic core and by strengthening hydrogen bonding at the membrane surface [reviewed in Chong (2010)].

Many studies have utilized high performance liquid chromatography-mass spectrometry (HPLC-MS) to investigate the presence of GDGTs in nature, including intact polar lipids and core lipids lacking the polar head group (Hopmans et al., 2000; Sturt et al., 2004). The intact polar lipids are typically attributed to living biomass, whereas the core lipids can accumulate in sediments and remain intact for hundreds of millions of years (White et al., 1979; Schouten et al., 2002; Sturt et al., 2004). These studies have led to the discovery of new GDGTs that were only later linked to specific microorganisms (Chappe et al., 1979; Michaelis and Albrecht, 1979; Sinninghe Damsté et al., 2000; Schouten et al., 2008) and established relationships between GDGT composition and environmental conditions. Reminiscent of patterns recognized in axenic cultures, several studies demonstrated a positive relationship between the number of GDGT cyclopentyl rings and seawater temperature (Schouten et al., 2000, 2002; Wuchter et al., 2004); however, studies in terrestrial geothermal environments have shown weaker relationships with temperature (Pearson et al., 2004, 2008; Zhang et al., 2006; Schouten et al., 2007b; Boyd et al., 2013).

Several biomarker proxies have been developed in accordance to the observation that iGDGT lipid composition in temperate environments is tied to physicochemical conditions. Investigations of modern sediments have shown that these biomarker proxies can be used to closely estimate paleoclimatic and paleoenvironmental conditions. For example, the TEX_86_ (TetraEther IndeX of tetraethers with 86 carbons) proxy uses iGDGT composition to estimate sea surface temperatures (Schouten et al., 2002, 2007a; Kim et al., 2009, 2010; Pearson and Ingalls, 2013). The methane index (MI) has been used to predict the occurrence of anaerobic oxidation of oxidation (AOM) by archaea, which occurs in association with cold seeps or areas containing gas hydrates (Zhang et al., 2011).

Despite a foundation based on studies of thermophile pure cultures and advances in understanding the environmental controls of iGDGT composition in temperate environments, the physicochemical controls of iGDGTs in terrestrial geothermal systems remain poorly understood (Pearson et al., 2004, 2008; Zhang et al., 2006; Schouten et al., 2007b; Pitcher et al., 2009; Boyd et al., 2013; Li et al., 2013). The objective of this study was to explore the geochemical correlates of the abundance and composition of iGDGTs in geothermal springs in the U.S. Great Basin. A companion paper on branched GDGTs from the same set of samples is published in the same issue of this journal (Hedlund et al., 2013).

## Materials and methods

### Sampling

**Figure 1:**
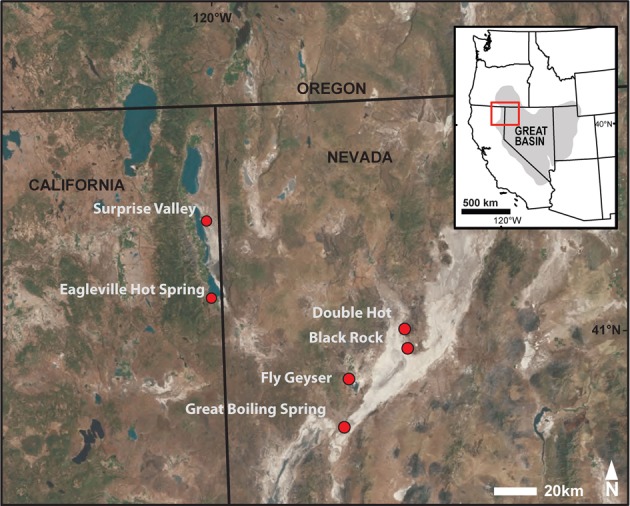
**Study sites located in the U.S. Great Basin within northwestern Nevada and northeastern California**.

Samples were collected from eight hot spring locations in the United States Great Basin, including sites in northwestern Nevada and northeastern California (Figure 1; Figure S2). Prior to sampling, temperature, pH, and conductivity were determined at the precise sampling location with a calibrated and temperature-corrected probe (LaMotte 5 Series, Chestertown, MD or YSI Model 30, Yellow Springs, OH and WTW Model pH330i, Weilheim, Germany). Sediment- or mat-water interface samples (about 1–2 cm) were collected with sterilized spoons, homogenized in sterilized pie tins, and transferred into four 50 mL Falcon tubes and ten 1.5 mL Eppendorf tubes. All samples were frozen immediately after collection and transported on dry ice before being stored at −80°C in the laboratory.

Water samples were collected for chemical analyses at each sampling location prior to mat or sediment sampling. Oxygen and sulfide were measured in the field using the HRDO Accuvac ampule method (Hach) and the Pomeroy methylene blue method (Hach), with modifications for high temperature as described (Miller-Coleman et al., 2012). Water samples for lab measurements were filtered using a 0.2 μm Pall Acrodisc® syringe filters, immediately frozen, and transported back to the lab for analysis by ion chromatography (anions (Br^−^, Cl^−^, F^−^, NO^−^_3_, NO^−^_2_, PO^−^_43_, SO^−^_42_), Dionex DX-500 chromatograph, AS14A column, with 10 μ M Na_2_CO_3_/NaHCO_3_ as an eluent, Dionex, USA) or by direct current plasma emission spectrometry (cations (Ca^2+^, Cr^3+^, Cu^2+^, Fe^3+^, K^+^, Mg^2+^, Mn^4+^, Na^+^, Ni^2+^, Sr^2+^, Zn^2+^), DCP-OES, Beckman, USA). NO^−^_3_, NO^−^_2_, and NH^+^_4_ were measured by automated colorimetry as described by Dodsworth et al. (2011a,b) (Lachat, USA).

### Lipid extraction

The sediment- or mat-water interface samples (about 1–2 cm deep) were collected with sterilized spoons, homogenized in sterilized pie tins, and transferred into 50 mL polypropylene tubes. All samples were frozen immediately after collection on dry ice and were stored at −80°C. In the laboratory, frozen samples were freeze-dried and powdered with a mortar and pestle. Lipids were extracted quantitatively from an initial mass of ~5 g of sediment/mat using a modified Bligh-Dyer extraction method (Lengger et al., 2012), consisting of four cycles of ultrasonication and centrifugation using a methanol:dichloromethane:phosphate buffer (2:1:0.8, v:v:v). The supernatants were collected and separation of the organic layer was achieved through the addition of 5 mL of dichloromethane (DCM) and 5 mL of deionized water (DIH_2_O). All extracts were evaporated under nitrogen. Dried lipids were dissolved in *n*-hexane:ethyl acetate (1:1, v:v) and MeOH as eluents to collect the non-polar fraction F1 (containing core GDGTs) and polar fraction F2 (containing polar GDGTs), respectively, via silica-gel column chromatography. After collection of the fractions, 30 μL of a GDGT C_46_ internal standard was added to all fractions. The polar fraction was divided into two parts (F2A and F2B). F2B was hydrolyzed with MeOH:HCl (95:5; v:v) and heated at 70°C for 3 h. Afterwards, the organic layer was extracted with the addition of DIH_2_O and DCM. An additional 1–3 mL of DCM was added to F2B to collect the organic layer. F1, F2A and F2B extracts were all dried under nitrogen and dissolved four times with *n*-hexane:isopropyl alcohol (99:1; v:v), filtered through a 0.45 μm polytetrafluoroethylene filter and dried under nitrogen. Lastly, dried lipids were dissolved once again with the addition of 600 μL of *n*-hexane:isopropyl alcohol (99:1; v:v) for analysis. F1 was run directly on LC-MS. F2A was also run directly to quantify core GDGTs that may be present in the polar fraction, which would be subtracted from F2B and added to F1.

### Liquid chromatography-mass spectrometry (LC-MS) and proxy calculations

All fractions were tested on an Agilent 1200 liquid chromatography equipped with an automatic injector coupled to QQQ 6460 MS and Mass Hunter LC-MS Manager software. Separation of peaks was achieved using a Prevail Cyano column (2.1 × 150 mm, 3 μm; Alltech, Deerfled, IL, USA) maintained at 40°C. The volume of injection was 5 μL. GDGTs were first eluted with 99% *n*-hexane and 1% isopropanol for 5 min, followed by a linear gradient to 1.8% isopropanol in 50 min at a constant rate of 0.2 ml/min (Li et al., 2013). Solvent was held for 7 min in 10% isopropanol and was then allowed to re-equilibrate in 1% isopropanol for 10min. Measurement of GDGTs was performed using Agilent 6460 triple-quadrupole MS with an atmospheric pressure chemical ionization (APCI) ion source. The scanning type used was single ion monitoring mode of protonated molecules. The conditions for APCI/MS were: nebulizer pressure 40 psi, vaporizer temperature 350°C, drying gas (N_2_) flow 5 L/min and temperature at 250°C, capillary voltage 3 kV, and corona 4 μA. All samples were quantified by integration of the peak area of [M+H]^+^ ions in the extracted ion chromatogram, and comparison to the C_46_ internal standard. The detection limit was 0.8 pg.

RI was calculated according to Schouten et al. (2007a), TEX_86_ according to Schouten et al. (2002), and MI according to Zhang et al. (2011):

$$\begin{array}{l} RI=\frac{\begin{array}{c} \text{GDGT}-\text{1 }+\text{ 2GDGT}-\text{2 }+\text{3GDGT}-\\ \text{3 }+\text{ 4GDGT}-\text{4 }+\text{ 5GDGT}-\text{5 }+\text{ 6GDGT}-\text{6} \end{array}}{\begin{array}{c} \text{GDGT}-\text{0 }+\text{ GDGT}-\text{1 }+\text{ GDGT}-\text{2 }+\text{ GDGT}-\text{3}\\ +\text{ GDGT}-\text{4 }+\text{ GDGT}-\text{5 }+\text{ GDGT}-\text{6} \end{array}}\\ MI=\frac{\text{GDGT}-\text{1 }+\text{ GDGT}-\text{2 }+\text{ 3GDGT}-\text{3}}{\begin{array}{c} \text{GDGT}-\text{1 }+\text{ GDGT}-\text{2 }+\text{ GDGT}-\text{3}\\ +\text{ crenarchaeol }+{\text{ crenarchaeol}}^{\prime } \end{array}} \end{array}$$

where GDGT-0 to GDGT-8, crenarchaeol and crenarchaeol' refer to GDGT structures in Figure S1.

### Statistical analyses

Statistical analyses are based upon relative abundance of individual lipids, unless otherwise noted. Relative abundance was calculated by dividing the absolute abundance of a given lipid by the total absolute abundance of iGDGTs measured in that sample. Absolute and relative abundances were calculated for core, polar, and core plus polar fractions, respectively.

Two-way cluster analyses were completed in PC-ORD (MjM Software Design) and modified from the clustering methodology in Pearson et al. (2008). Relative abundance data from the polar lipid fraction were imported into PC-ORD. Input data were relativized by the variable's maximum (as recommended by PC-ORD), and a dissimilarity matrix using Sørensen (Bray-Curtis) distance measures was computed. From the distance matrix an agglomerative hierarchical clustering tree was assembled using the flexible beta method (β = −0.25). In order to remove bias from incorrectly concluding the absence of an iGDGT that may occur below detection limits (see Pearson et al., 2008), samples with fewer than three iGDGT lipid types were removed from the cluster analysis. A second set of two-way cluster analyses were completed on the absolute abundance lipid data from hot spring and soil samples (polar fraction), using the clustering methodology described above.

Non-metric multidimensional scaling (NMS) analyses were completed in PC-ORD to explore multivariate relationships among lipids and geochemical analytes. NMS is ideally suited to ecological datasets as it does not assume linear relationships or normal distributions of data (McCune and Grace, 2002). The relative abundance data from the polar lipid fraction (same data from cluster analyses) were imported into PC-ORD. Lipid and associated geochemical data were relativized by the analyte's maximum prior to analyses. An ordination of iGDGTs was calculated using Sørensen (Bray-Curtis) distance measures in the NMS “autopilot mode” in PC-ORD. The NMS analyses included 100 initial runs, which were used to determine the optimal number of axes. Monte Carlo testing was completed to determine statistical significance and included 50 runs on actual data and 50 runs on randomized data. The final ordination was executed for the recommended number of axes using 99 runs. Ordinations of lipid data were plotted with geochemical analytes, to show correlations between the NMS model and physicochemical variables.

Bivariate and univariate analyses were completed to explore relationships of the core, polar, and core plus polar lipid data to geochemical analytes. Non-parametric analyses were chosen to avoid assumptions of normality and linear relationships between variables. Spearman's rho values were calculated in SPSS to identify correlative relationships between iGDGTs and geochemical analytes. Mann-Whitney *U*-tests were used to determine differences in lipid composition among select temperature classes. Parametric regression analyses were completed to explore linear relationships among select variables. Bivariate and univariate analyses were completed in SPSS (IBM SPSS Statistics) at the 0.05 level of significance.

## Results

### Spring geochemistry

Forty hot spring sediment and microbial mat samples were collected from eight different U.S. Great Basin locations, with temperatures ranging from 31 to 95°C and pH values from 6.8 to 10.7 (Table 1). The hottest samples were collected from high-temperature geothermal sources reaching 95°C. Cooler samples were collected from outflow channels and some warm spring sources. The springs sampled here were all circumneutral to alkaline but included both Na-Cl-type springs (Great Boiling Spring, Sandy's Spring West, Rick's Hot Creek) and Na-HCO_3_-Cl-type springs (Fly Geyser, Surprise Valley, Eagleville) (Anderson, 1978; Supplementary Table 1).

**Table 1:** **Isoprenoidal GDGT absolute abundance, hot spring temp and pH**.

**Hot spring area**	**Sampling location**	**Temp (°C)**	**pH**	**Absolute abundance of core + polar iGDGT (ng of lipid/g dry mass)**												
				**iGDGT-0 m/z 1302**	**iGDGT-1 m/z 1300**	**iGDGT-2 m/z 1298**	**iGDGT-3 m/z 1296**	**iGDGT-4 m/z 1294**	**iGDGT-5 m/z 1292**	**Crenarchaeol m/z 1292′**	**Cren Isomer m/z 1292″**	**iGDGT-6 m/z 1290**	**iGDGT-7 m/z 1288**	**iGDGT-8 m/z 1286**	**Total iGDGT**	**% Polar iGDGTs**
**GREAT BOILING SPRINGS**																
Great boiling spring	GBS 19	95.0	6.80	276	214	229	257	761	155	34.8	BDL	5.04	3.74	2.45	1940	60
	GBS A	81.0	7.41	8.76	4.28	5.31	6.09	8.68	0.52	BDL	BDL	BDL	BDL	BDL	33.6	35
	GBS B	78.0	7.35	11.0	6.45	10.5	11.7	16.4	3.33	0.34	0.46	0.17	0.21	BDL	60.6	80
	GBS C	65.0	7.69	64.9	46.2	76.5	81.2	102	28.1	1.62	BDL	BDL	BDL	BDL	401	62
	GBS 61—Y	61.0	8.00	70.5	33.7	53.0	65.8	63.8	31.5	33.5	BDL	BDL	BDL	BDL	352	52
Sandy's springs west	SSW SOURCE	80.0	7.37	6.05	1.17	1.62	1.46	2.40	4.34	0.54	BDL	0.13	0.28	BDL	18.0	0.00
	SSW 70	70.0	7.86	21.4	14.0	24.8	10.1	6.50	34.2	2.53	BDL	1.54	3.82	BDL	119	58
	SSW 60	60.0	7.90	43.3	17.1	17.8	4.36	BDL	146	17.7	BDL	1.19	0.69	BDL	248	40
	SSW 50 (OF)	51.0	8.17	^1^BDL	BDL	BDL	0.51	0.39	17.4	1.16	BDL	BDL	BDL	BDL	19.4	23
	SSW 40	39.0	8.49	19.9	10.6	12.8	4.69	5.58	142	14.3	BDL	BDL	BDL	BDL	210	40
Rick's hot creek	RHC 5	90.0	7.49	21.7	8.69	12.7	18.3	29.0	1.35	0.25	0.38	0.09	BDL	BDL	92.5	42
	RHC 4	79.0	7.75	3.28	1.19	1.81	1.89	4.36	1.14	0.16	BDL	BDL	BDL	BDL	13.8	38
	RHC 3	69.0	7.96	3.40	1.42	1.83	2.02	2.52	5.91	0.36	BDL	0.02	0.04	BDL	17.5	82
	RHC 2	62.0	8.23	5.96	1.43	2.17	3.76	4.28	6.99	0.57	0.24	0.01	0.01	BDL	25.4	81
	RHC 1	52.0	8.43	36.4	7.18	16.6	20.9	29.1	29.9	3.15	BDL	0.30	0.30	BDL	144	27
**OTHER BLACK ROCK DESERT SPRINGS**																
Fly geyser	FG 60	60.0	8.37	BDL	BDL	BDL	BDL	BDL	BDL	BDL	BDL	BDL	BDL	BDL	BDL	^2^N/A
	FG 50	50.0	8.60	0.21	0.02	0.04	0.01	0.03	0.15	0.01	BDL	BDL	BDL	BDL	0.47	39
	FG 40	42.0	8.80	BDL	BDL	BDL	0.01	BDL	0.01	0.00	BDL	BDL	BDL	BDL	0.02	0.00
	DH SOURCE	80.0	8.05	0.77	0.56	0.93	2.26	2.52	2.22	0.20	BDL	BDL	BDL	BDL	9.46	30
	DH 70	70.0	8.37	8.90	4.89	10.8	2.89	1.80	19.1	1.41	BDL	0.48	1.18	BDL	51.4	49
	DH 60	60.0	8.74	2.20	0.86	2.08	0.99	1.14	17.7	1.39	0.21	0.34	0.68	BDL	27.6	26
	DH SOURCE 2	55.0	8.50	2.34	0.70	0.91	0.86	0.90	2.51	0.18	BDL	BDL	BDL	BDL	8.39	35
	DH 50	50.0	9.09	8.88	BDL	BDL	BDL	BDL	3.22	0.12	BDL	BDL	BDL	BDL	12.2	75
	DH 43	44.0	9.24	4.56	1.04	1.72	0.75	0.80	9.31	0.67	BDL	BDL	BDL	BDL	18.8	38
	DH 31	31.0	10.70	0.12	0.03	0.02	BDL	0.03	0.17	BDL	BDL	BDL	BDL	BDL	0.36	0.00
Black rock	BR 45	50.0	7.88	32.7	9.04	10.5	0.77	0.36	0.79	0.04	BDL	BDL	BDL	BDL	54.2	78
**SURPRISE VALLEY SPRINGS**																
Eagleville	EV 44	44.0	9.73	1.13	1.07	0.81	0.72	1.13	36.1	2.72	BDL	0.20	BDL	BDL	43.9	30
	EV 43	44.0	9.73	11.7	9.23	10.7	3.04	4.10	112	9.04	BDL	0.78	0.41	BDL	161	24
Surprise valley	SV SOURCE	86.0	8.34	7.11	8.87	12.9	18.1	19.6	4.35	0.70	BDL	BDL	BDL	BDL	71.7	45
	SV 70	69.0	8.50	7.44	4.49	7.06	4.42	3.49	4.01	0.48	BDL	BDL	0.08	BDL	31.5	55
	SV 60	60.0	8.64	8.25	4.84	5.13	2.39	1.05	6.40	0.66	BDL	0.03	0.10	BDL	28.9	55
	SV 50	49.0	8.78	14.5	6.91	8.35	5.30	4.39	14.8	1.36	BDL	0.26	0.33	BDL	56.2	41
	SV 40	41.0	9.07	7.36	1.96	3.93	0.68	0.57	1.22	0.11	BDL	BDL	BDL	BDL	15.8	45
	SVX SOURCE	84.0	8.41	184	113	156	182	167	59.6	10.7	BDL	BDL	BDL	BDL	870	0.70
	SVX 70	69.0	8.58	6.59	2.80	4.95	2.35	1.55	6.93	0.61	BDL	BDL	BDL	BDL	25.8	54
	SVX 60	61.0	8.72	4.64	1.85	2.04	1.73	1.14	6.36	0.78	BDL	BDL	BDL	BDL	18.5	50
	SVX 50	50.0	8.98	1.94	1.17	1.21	0.58	0.78	3.83	0.20	BDL	BDL	BDL	BDL	9.70	41
	SVX 2	83.0	8.24	7.21	5.71	8.18	9.39	17.5	27.3	2.14	BDL	0.20	BDL	BDL	77.6	43
	SVX 1	77.0	8.32	5.11	3.12	5.40	6.17	6.14	2.31	0.10	BDL	BDL	BDL	BDL	28.4	36
	SVX 3	41.0	8.22	8.01	2.43	2.31	1.61	1.53	1.06	0.28	BDL	BDL	BDL	BDL	17.2	0.00

1 BDL, below detection limit; detection limit for polar lipids is 0.8 pg.

2 N/A, percent polar lipids not available because total polar + core iGDGTs were below detection limit.

### iGDGT abundance

**Figure 2:**
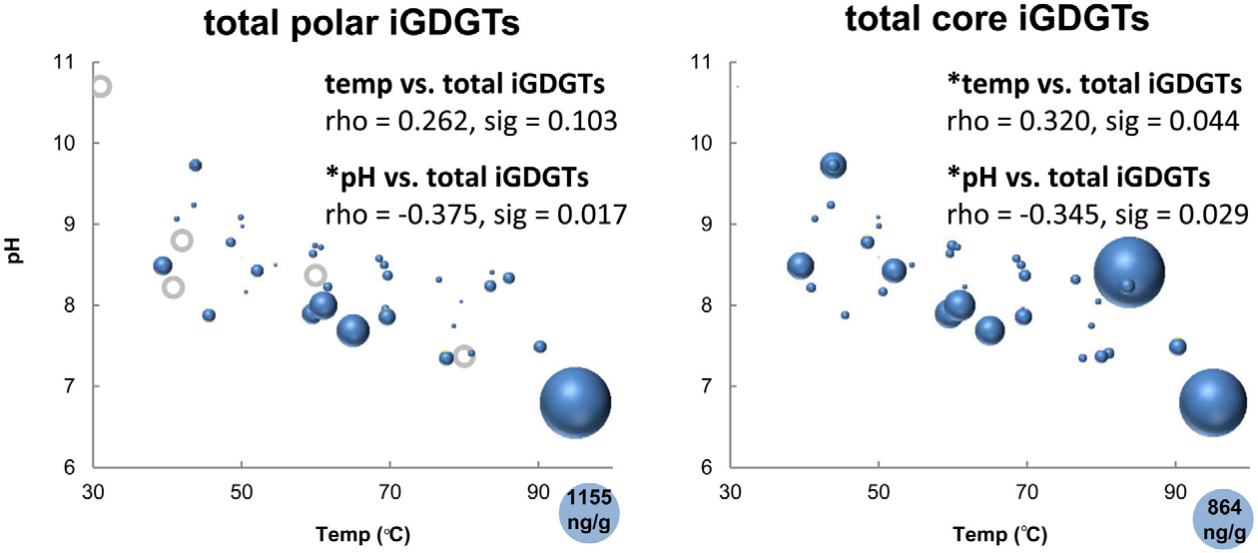
**Bubble plots showing the absolute abundance core and polar iGDGTs as a function of temperature and pH.** Areas of bubbles are scaled to the absolute abundance of iGDGTs in ng lipid per gram dry mass. Gray circles represent zero abundance. Correlations between temperature, pH and total iGDGTs are reported as Spearman's rho values. Maximum relative abundances are shown in the lower right corner of each plot.

**Figure 3:**
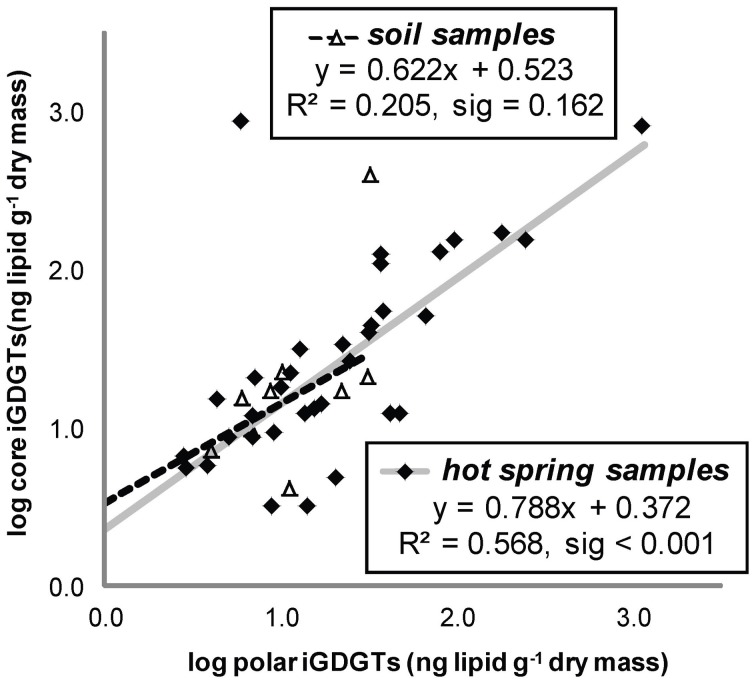
**Absolute abundance of polar iGDGTs vs. core iGDGTs for soil and hot spring samples.** Samples below the method detection limit for polar iGDGTs are not shown and were not used for in regression analyses (5 hot spring, 2 soil). Samples with <1 ng/g of polar or core iGDGTs, with negative log10 values are not shown; however, these values were used for regression analyses (1 hot spring, 2 soil).

All hot spring samples except one site at Fly Geyser (60°C) contained measureable concentrations of iGDGTs (Table 1). Absolute abundance of polar iGDGTs ranged from below detection limit to 1155 ng lipid g^−1^ dry mass. Concentrations of core iGDGTs ranged from below detection limit to 864 ng lipid g^−1^ dry mass (Table 1; Figure 2). The absolute abundance of both polar and core iGDGTs was negatively correlated with pH; core iGDGT abundance was also positively correlated with temperature (Figure 2). Core and polar iGDGTs in hot spring samples were highly related, as shown by a linear regression analysis of log-transformed absolute abundances of the polar vs. core iGDGTs (Figure 3; *R*^2^ = 0. 568; sig < 0.001).

**Figure 4:**
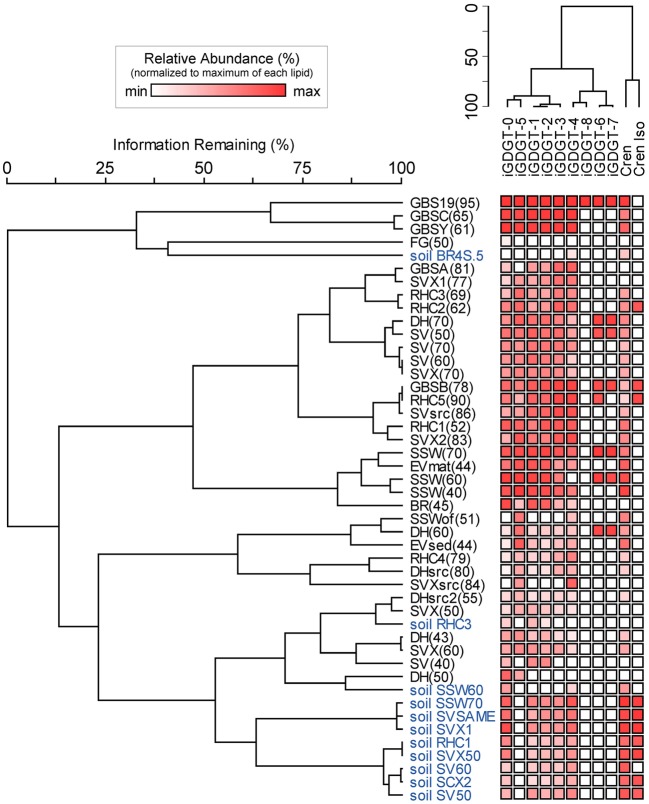
**Two-way cluster analysis based on the absolute abundance of polar iGDGTs in soil (blue) and hot spring (black) samples.** The horizontal dendrogram groups samples according to similarity in iGDGT composition. The vertical dendrogram groups samples according to iGDGT presence and abundance. Heat map colors indicate minimum (white) to maximum (red) abundance of iGDGT types, wherein each lipid is scaled to its maximum absolute abundance (reported as ng lipid per g dry mass): iGDGT-0 (156.1), iGDGT-1 (155.2), iGDGT-2 (150.9), iGDGT-3 (144.8), iGDGT-4 (420.8), iGDGT-5 (110.5), crenarchaeol (11.3), cren isomer (1.3), iGDGT-6 (3.4), iGDGT-7 (2.8), iGDGT-8 (1.9).

Soil samples were collected in the vicinity of the hot springs in order to help assess whether iGDGTs in the hot springs may derive from soil microorganisms (Supplementary Table 1). In contrast to hot spring samples, core and polar iGDGTs in soil samples were moderately related, as indicated by the weak positive relationship between log transformed polar vs. core lipid fractions (Figure 3; *R*^2^ = 0.205, sig = 0.162). The range of polar iGDGT concentrations in hot spring samples was much wider than the soil samples and some hot spring samples had a >50-fold higher concentration of polar iGDGTs than the highest concentrations measured in soils (Figure 3). In addition, a two-way cluster analysis was created to group samples according to the absolute abundance of iGDGTs along one axis and iGDGTs according to their co-distribution within samples along a second axis (Figure 4). This revealed that iGDGT profiles in soil samples were distinct from most hot spring samples (Figure 4). In particular, polar iGDGT-5, -6, -7, and -8 were only found in hot springs and were absent in soils. In contrast, polar crenarchaeol and crenarchaeol regio-isomer were found in most soil samples at absolute concentrations exceeding their concentrations in most hot spring samples. Polar iGDGT-0, -1, -2, -3, and -4 were all common in both hot spring and soil samples at a wide range of concentrations; however, the highest concentrations of all five lipids were found in hot springs. Significant linear relationships between log-transformed polar and core lipids for iGDGT-0, -1, -3, -4, -5 and crenarchaeol were only observed for hot spring samples. A positive linear relationship between log transformed polar and core iGDGT-2 was significant in both soil and hot spring samples (Figure S3).

### Relationship of iGDGTs to geochemistry

**Figure 5:**
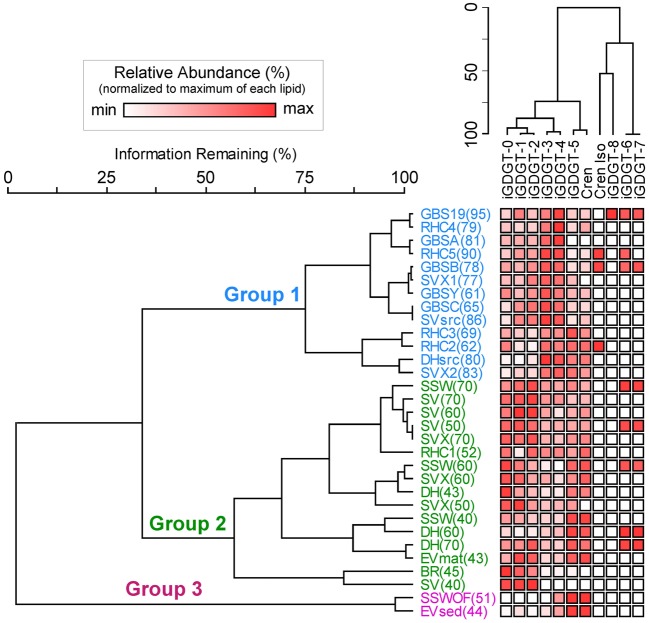
**Two-way cluster analysis based on the relative abundance of polar lipids in hot spring samples.** The horizontal dendrogram groups samples according to similarity in polar iGDGT composition. The vertical dendrogram groups samples according to iGDGT presence and abundance. Heat map colors indicate minimum (white) to maximum (red) abundance of iGDGT types, wherein each lipid is scaled to its maximum contribution to the total iGDGTs found in a sample. Maximum relative abundance (%) for each lipid is as follows: iGDGT-0 (100.0), iGDGT-1 (23.9), iGDGT-2 (43.3), iGDGT-3 (24.1), iGDGT-4 (73.8), iGDGT-5 (77.2), crenarchaeol (14.8), cren isomer (1.1), iGDGT-6 (1.7), iGDGT-7 (2.9), iGDGT-8 (0.2).

A second two-way cluster analysis of the polar lipid fraction was completed for hot spring sediment and mat samples to assess lipid co-occurrence in the samples and to group samples according to the relative abundance of lipids (Figure 5). Mat and sediment samples were clustered into three primary groups, Group 1, Group 2, and Group 3, which varied according to temperature. Group 1 was comprised primarily of high-temperature samples (≥61°C) and contained elevated relative abundances of iGDGT-3 and iGDGT-4. Within Group 1, samples from individual springs clustered together to some extent, especially samples from Great Boiling Spring (GBS) and Rick's Hot Creek (RHC). In contrast, the lower to mid-range temperature samples (40–70°C) comprising Group 2 contained some of the highest relative abundances of iGDGT-0, iGDGT-1, and iGDGT-2. Within Group 2, samples from Surprise Valley (SV) clustered together to some extent. Samples in Group 3 contained highest relative abundances of iGDGT-5 and crenarchaeol and included two low-temperature sites (44 and 51°C).

iGDGT types clustered according to the number of cyclopentyl rings into the following groups: (1) iGDGTs with 0–4 cyclopentyl rings, (2) iGDGT-5 and crenarchaeol, (3) cren isomer and iGDGT-8, and (4) iGDGTs with 6 and 7 cyclopentyl rings. iGDGT-8 only occurred in GBS 19, which also had the highest temperature (Table 1).

**Figure 6:**
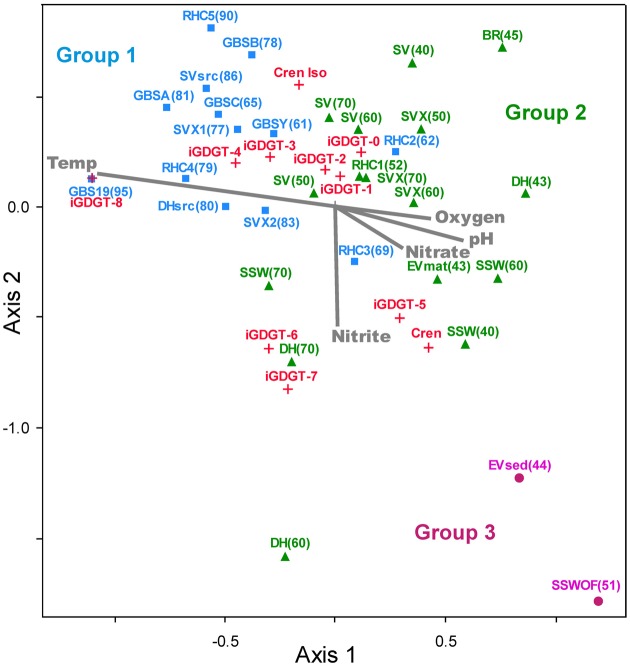
**The NMS plot shows relationships among lipids and environmental variables.** Distance on the plot is proportional to dissimilarity in lipid composition (relative abundance). Samples sites (solid shapes) are arranged accordingly to similarity in polar iGDGT composition. Lipids (red +) appear where the centroid of that variable would be placed within the NMS model. Environmental variables (gray) appear as vectors that indicate relative correlation with NMS axes. Colors of samples sites correspond to groups identified in the two-way cluster analysis.

Non-metric multidimensional scaling (NMS) plotted hot spring sediment and mat samples according to the relative abundance of polar iGDGTs (Figure 6). The 3-axis NMS model indicated strong underlying structure, which resulted in a low-stress and statistically significant ordination (minimum stress = 6.638, *p* = 0.0196). The NMS model was combined with overlays of individual iGDGTs and environmental vectors. Among the environmental variables, temperature showed the strongest correlation with the NMS model (*r*^2^ = 0.546 correlation to axis 1, Supplemental Table 2).

A few other analytes also correlated to NMS axes (Supplemental Table 2), including pH (*r*^2^ = 0.359, axis 1), chloride (*r*^2^ = 0.330, axis 3), nitrite (*r*^2^ = 0.286, axis 2), oxygen (*r*^2^ = 0.259, axis 1), and nitrate (*r*^2^ = 0.215, axis 1). Spearman's rho analyses also indicated correlations among individual lipids and these analytes. For example, polar fractions of iGDGT-3 and iGDGT-4 were negatively associated with pH (rho = −0.485, sig = 0.002; rho = −0.502, sig = 0.001), nitrate (rho = −0.430, sig = 0.007; rho = −0.437, sig = 0.006), and chloride (rho = −0.387, sig = 0.016; rho = −0.431, sig = 0.007) (see complete correlation dataset in Supplementary Table 3).

### Evidence of temperature optima for iGDGTs

**Figure 7:**
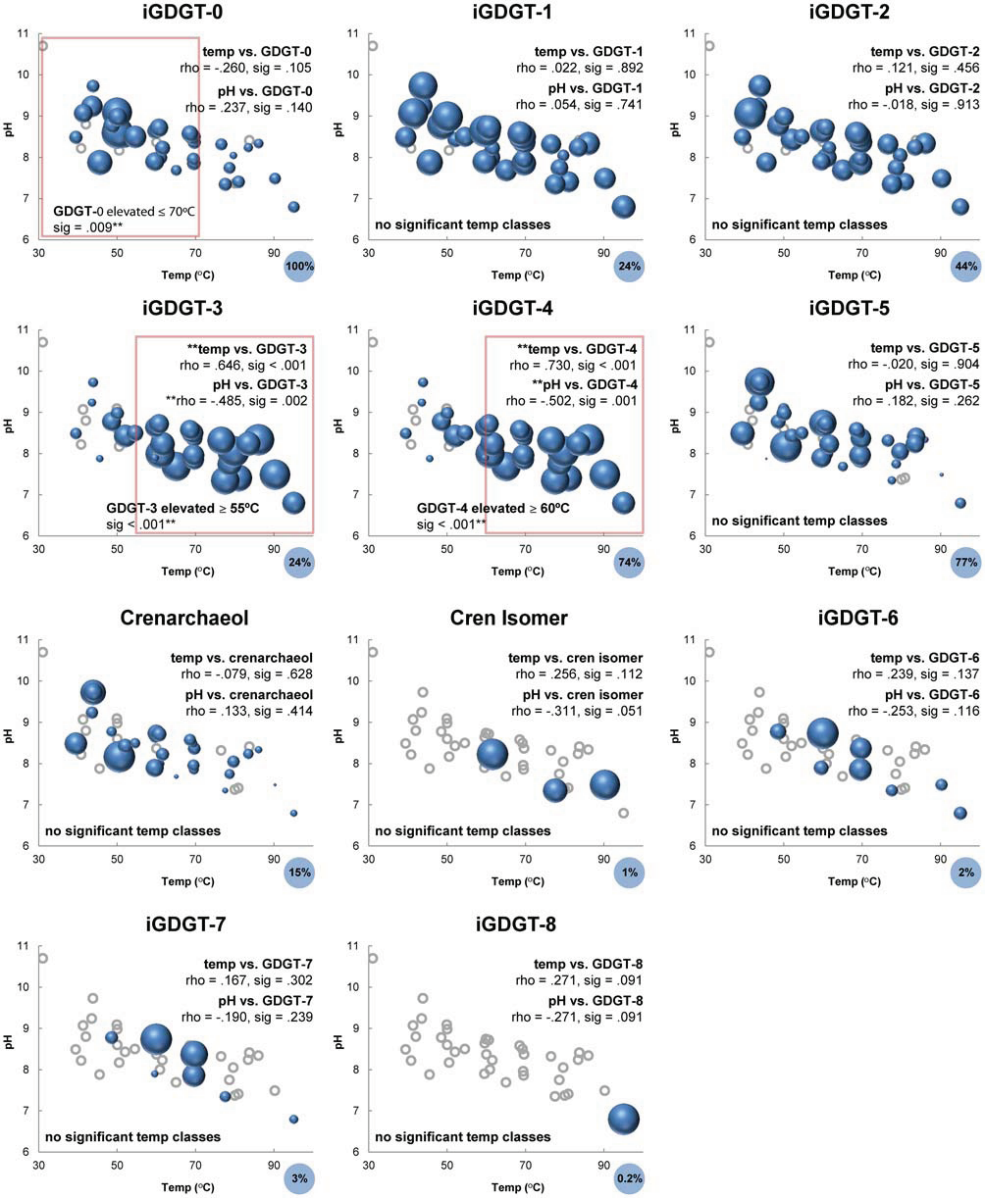
**Bubble plots showing relative abundance of polar iGDGTs types as a function of temperature and pH.** Areas of bubbles are scaled to the maximum relative abundance observed for of that lipid type. Gray circles represent zero abundance. Correlations between temperature, pH and iGDGT types are reported as Spearman's rho values. Temperature optima classes of iGDGT types were tested using Mann-Whitney tests. Maximum relative abundance of each lipid (%) is indicated in lower right corner of the plots.

The relative abundance profiles of iGDGT types, particularly polar lipids, further illustrated the variable distribution of lipids across the temperature/pH gradient (Figure 7). As observed in the two-way cluster analysis and NMS, the relative abundance of iGDGTs in the temperature/pH gradient was defined by the number of cyclopentyl rings (Figure 7). iGDGT-0 contains no cyclopentyl rings and had highest relative abundance at temperatures ≤70°C (Mann-Whitney test, sig = 0.009). iGDGT-1 and iGDGT-2 had fairly uniform relative abundances across the temperature/pH gradient, as supported by a lack of significant temperature relationships observed in Spearman's rho correlation coefficients and Mann-Whitney tests (comparing lipid abundance above and below the median temperature of 63°C). In contrast, both iGDGT-3 and iGDGT-4 were positively correlated with temperature (rho = 0.646, sig < 0.001; rho = 0.730, sig < 0.001), and were significantly more abundant at temperatures at or above 55 and 60°C, respectively (Mann-Whitney tests, sig < 0.001). Weak relationships existed between temperature and the abundance of iGDGTs with five or more rings, as indicated by a lack of significant relationships between temperature and crenarchaeol, iGDGT-5, iGDGT-6, and iGDGT-7.

### Indices and bioindicators

Spearman's rho and regression analyses indicated a few statistically significant relationships between geochemical variables and iGDGT-based indices or bioindicators (Supplementary Table 2). The ring index (RI) based on polar lipids was positively correlated to temperature (rho = 0.425, sig = 0.011), which corresponded to a weak linear regression (*r*^2^ = 0.107, *p* = 0.055). The TEX_86_ index of polar iGDGTs was also positively associated with temperature (rho = 0.618, sig. < 0.001), which reflected a moderate linear relationship (*R*^2^ = 0. 367, sig < 0.001). The methane index (MI) of polar iGDGTs was negatively associated with nitrate (rho = −0.448, sig = 0.012) but positively associated with temperature (rho = 0.361, sig = 0.042).

## Discussion

### Evidence of *in situ* iGDGT production

Lipid data indicated that most, if not all, iGDGTs are produced within the hot springs. The absolute abundance of polar and core iGDGTs were highly correlated, which has been interpreted as evidence of production and subsequent degradation of iGDGTs within an environment (Liu et al., 2011). This relationship also held for analyses of most individual lipids in hot spring samples. In contrast, the linear relationship of log-transformed core and polar iGDGTs was weak for soil samples, and this positive relationship was dominated by iGDGT-2. Polar crenarchaeol, iGDGT-0, and possibly iGDGT-2 appear to be produced in Great Basin desert soils, as indicated by high absolute abundance and regression analysis results. Crenarchaeol and iGDGT-0 are both produced by AOA, but the latter is also produced by methanogens (see discussion below). These data suggest all iGDGTs examined in this study are produced in Great Basin geothermal springs, and there is no strong evidence of contamination of geothermal samples with lipids produced in nearby soils.

The sample collected from the highest temperature source in the Great Boiling Spring area, GBS-19 (95°C), appears to be a significant outlier, because the absolute concentration of polar iGDGTs was four times higher in this sample than any other sample (Figure 2) and this was the only sample in which iGDGT-8 was detected.

### Temperature relationships

While several geochemical analytes correlated with individual lipids in this study, the data suggest temperature may be a primary driver of lipid composition in these hot spring sediments and mats. Temperature showed the strongest correlation to the lipid NMS model (*r*^2^ = 0.546 correlation to axis 1), as indicated by the length of its vector (Figure 5). Other analytes, including pH, nitrite, oxygen, and nitrate also showed strong correlations with the NMS model (Figure 5; Supplemental Table 2) and bivariate correlations with individual iGDGTs (Supplemental Table 3); however, these relationships may reflect co-variation of these geochemical analytes with temperature. Alkaline geothermal springs commonly exhibit elevated temperatures and reduced conditions at their sources, giving rise to higher pH and more oxidized conditions as they cool (Nordstrom et al., 2005). For example, the speciation of nitrogen often changes from ammonia/ammonium to nitrate along hot spring outflow channels due to the activity of thermophilic AOA and nitrite-oxidizing bacteria (Dodsworth et al., 2011b; Holloway et al., 2011; Edwards et al., 2013). Oxygen concentration and pH are controlled in hot spring outflow systems by gas exchanges with the atmosphere. Oxygen solubility is inversely proportional to temperature and increases as geothermal water interacts with the atmosphere. In contrast, CO_2_ is lost along outflow channels due to degassing and autotrophy, causing pH to increase (Nordstrom et al., 2005). The NMS environmental overlay illustrates these phenomena and shows the negative association of pH, nitrite, oxygen, and nitrate to temperature (Figure 6).

Other lipid surveys in terrestrial hot springs have reported little direct correlation between the number of cyclopentyl rings and temperature (Pearson et al., 2004, 2008; Schouten et al., 2007b). Although temperature had a strong effect on the relative abundance of lipids sampled in this study, the relationship between temperature and iGDGT cyclization was not simple, as can be seen by the location of different iGDGTs on the NMS plot (Figure 6). As suggested previously (Schouten et al., 2007a,b), the more complex relationship between temperature and cyclization in geothermal environments largely reflects wholesale changes in microbial communities, driven by steep gradients in geochemical conditions such as temperature, redox status, and pH. This contrasts with environments that are relatively stable, spatially and temporally, such as the marine water column, which apparently hosts a much lower diversity of iGDGT-producing archaea that physiologically modulate iGDGT cyclization in response to comparatively minor temperature differences. The complex relationship between temperature and iGDGTs in this study is exemplified by the positive correlation between temperature and the RI of the polar lipids (rho = 0.425, sig = 0.011), but a weak linear regression relating RI to temperature (*r*^2^ = 0.107, sig = 0.055). Within our dataset, iGDGTs with 0–4 cyclopentyl rings follow the expected temperature trend of the RI to some extent (Figures 6, 7). For example, the apparent temperature optima for iGDGT-0 (abundant ≤ 70°C), iGDGT-3 (abundant ≥ 55°C), and iGDGT-4 (abundant ≥ 60°C) do show a general trend toward a higher degree of cyclization at higher temperatures.

### Possible source organisms and relationship to temperature

Although 16S rRNA gene surveys were not analyzed concomitantly with lipids, a number of studies on the distribution of terrestrial thermophiles in Great Basin hot springs identify possible sources of the iGDGTs studied here. Most Euryarchaeota produce iGDGT-0 as the only iGDGT, including all methanogen orders common in terrestrial environments, Methanosarcinales, Methanomicrobiales, Methanobacteriales, and Methanococcales (reviewed in Pearson and Ingalls, 2013; Schouten et al., 2013). Two of these orders, Methanomicrobiales and Methanobacteriales, have been detected in Great Basin hot springs by 16S rRNA gene sequencing in samples that were 67 and 56°C; these samples also hosted Thaumarchaeota and the yet-uncultivated Miscellaneous Crenarchaeotal Group I (MCG) but not the Thermoprotei (Crenarchaeota) (Huang et al., 2007). However, a number of other cultivation-independent studies in higher temperature Great Basin hot springs failed to detect significant numbers of methanogen sequences, including several deep sequencing efforts employing pyrotag sequencing (Pearson et al., 2004; Costa et al., 2009; Dodsworth and Hedlund, 2010; Dodsworth et al., 2011b; Cole et al., 2013; Peacock et al., 2013). Thus, the apparent temperature optimum of ≤70°C for iGDGT-0 is consistent with the absence or very low abundance of methanogens in Great Basin hot springs ≥70°C. This temperature is also consistent with the temperature limit observed for methanogenesis using enrichment culture at Yellowstone National Park, which was 72°C (Zeikus et al., 1980). Polar iGDGT-0 at higher temperatures, albeit at low relative abundance, could derive from a large number of microorganisms, but may possibly be connected with the presence of *Archaeoglobus*, which is known to produce iGDGT-0 as the only iGDGT (Trincone et al., 1992). Several studies have documented *Archaeoglobus* in Great Basin hot springs at temperatures up to 85°C (Costa et al., 2009; Dodsworth et al., 2011b; Cole et al., 2013; Peacock et al., 2013).

Crenarchaeol is a likely biomarker for AOA in natural environments (Leininger et al., 2006), including terrestrial geothermal environments (Pearson et al., 2004, 2008; Zhang et al., 2006; de la Torre et al., 2008; Pitcher et al., 2009, 2010; He et al., 2012; Boyd et al., 2013). Most Thaumarchaeota also produce the crenarchaeol regio-isomer and iGDGT-0, -1, -2, -3, and -4 (Schouten et al., 2013). Studies of laboratory cultures demonstrated that *Nitrososphaera gargensis* and “*Ca*. Nitrosocaldus yellowstonii” produce crenarchaeol up to at least 46 and 72°C, respectively (de la Torre et al., 2008; Pitcher et al., 2010). However, polar crenarchaeol has been observed previously in hot springs reaching 89°C (Pitcher et al., 2009). We detected polar crenarchaeol in two samples >90°C, Rick's Hot Creek (RHC 5, 90.2°C) and the highest temperature source in the Great Boiling Spring system (GBS-19; 95.0°C); however, we urge caution in using these low concentrations of polar crenarchaeol to interpret that AOA can grow in Great Basin hot springs at these temperatures, particularly due to the steep geothermal gradient at GBS-19. A very high abundance of crenarchaeol was recovered from a Surprise Valley spring (SVX source, 83.7°C), which is close to the *in situ* temperature limit for “*Ca*. Nitrosocaldus yellowstonii” apparent from 16S rRNA gene pyrotag data (~82°C; Cole et al., 2013) and the temperatures at which ammonia oxidation has been measured in Great Basin springs (~82°C; Dodsworth et al., 2011b) and Icelandic springs (85°C; Reigstad et al., 2008). Our analyses indicate no statistically significant relationships between the relative abundance of polar crenarchaeol and temperature. A large number of springs sampled in this study lacked measureable polar crenarchaeol, but among those sites that contained this lipid, crenarchaeol relative abundance was elevated below 70°C (Figure 6), which is generally consistent with the optimal temperature of 40°C proposed for crenarchaeol abundance relative to other iGDGTs (Zhang et al., 2006). In our study, the relative abundance of polar crenarchaeol was positively correlated with nitrite (rho = 0.343, sig = 0.035), which is a product of ammonia oxidation that signals an active oxidative nitrogen cycle, as has also been observed in studies of iGDGTs in Tibetan hot springs (Li et al., 2013).

As discussed above, the relationships between iGDGT-3 (abundant ≥ 55°C) and iGDGT-4 (abundant ≥ 60°C) are consistent with an increase in cyclization at high temperature, which has been observed for a number of thermophiles (Gliozzi et al., 1983; reviewed in Chong, 2010). These lipids, along with iGDGT-0, -1, and -2, are synthesized by a variety of thermophilic lineages that have been detected in Great Basin hot springs, such as Thermoproteales, Desulfurococcales, and relatives of *Aciduliprofundum* (Pearson et al., 2004; Costa et al., 2009; Dodsworth and Hedlund, 2010; Dodsworth et al., 2011b; Cole et al., 2013; Peacock et al., 2013). The sources of iGDGT-5, -6, -7 and -8 are more enigmatic. These lipids are produced by the acidophilic orders Thermoplasmatales (Langworthy et al., 1983; Macalady et al., 2004) and Sulfolobales (De Rosa and Gambacorta, 1988; Sturt et al., 2004) and by a few thermoacidophilic Thermoproteales (Thurl and Schäfer, 1988; Boyd et al., 2011). The acidic conditions favoring these highly cyclized iGDGTs are not known to exist in the Great Basin (Zehner et al., 2006) and, with the exception of *Thermoproteus*, which can synthesize iGDGT-5, no archaea known to synthesize iGDGT-5, -6, or -7 have been detected in Great Basin hot springs. iGDGT-5 was widely distributed in Great Basin hot springs in relatively high concentrations. iGDGT-6 and iGDGT-7 were found sporadically at low concentrations but were commonly co-located, suggesting a single source for those two lipids in Great Basin springs or a common geothermal environment favoring growth of different archaea making those two lipids. The low abundance of iGDGTs with 6–8 cyclopentyl rings in neutral to alkaline terrestrial geothermal systems has been observed previously (Li et al., 2013).

### Environmental proxies

TEX_86_ (TetraEther indeX containing 86 carbon atoms) is an index that commonly displays a strong positive correlation with temperature within aqueous environments. Calibration of TEX_86_ makes it a useful paleothermometer for estimating ancient surface water temperatures of the ocean (Schouten et al., 2002) and lakes (e.g., Blaga et al., 2009; Powers et al., 2010). In geothermal systems, TEX_86_ is mainly used to understand the influence temperature has on cyclization of iGDGTs. For example, Pearson et al. (2004) investigated a variety of hot springs from Nevada and California, and data from that study indicated TEX_86_ was not strongly related with temperature but positively correlated with HCO^−^_3_. The work by Li et al. (2013) on Tibetan hot springs indicated a negative correlation between TEX_86_ and temperature, which was contrary to the relationships observed in marine environments. Spearman's rho analyses of our dataset showed a positive correlation between temperature and TEX_86_ based on polar iGDGTs (rho = 0.618; sig. < 0.001); however, regression analyses showed a weak linear relationship (*R*^2^ = 0.367, sig < 0.001) that would be difficult to directly transfer to a temperature proxy. Given disparate results from our work and a variety of other studies, it seems clear that TEX_86_ cannot be used as a paleothermometer in geothermal environments. As such, it is important to rule out geothermal influence on sediments as a prerequisite for use of TEX_86_ as a paleothermometer. As discussed above and by others (Schouten et al., 2007a), the much wider range of geochemical conditions in geothermal systems, compared to marine and lacustrine environments where TEX_86_ has proven useful, translates into complex and less predictable community responses, rather than physiological responses that are assumed to be the basis for the TEX_86_ paleothermometer in temperate systems.

Although methane index MI has been used to predict the occurrence of anaerobic methane oxidation (AOM) by archaea in cold seeps or gas hydrates in marine environments (Zhang et al., 2011), it also has been used to infer AOM in association with denitrification processes in Tibetan hot springs (Li et al., 2013). Our results show a negative correlation between nitrate and the MI (rho = −0.445, sig = 0.014), similar to the observation made by Li et al. (2013).

## Conclusion

Several lines of evidence indicate temperature and/or pH may be the primary physicochemical controls of *in situ* iGDGT production in the hot spring systems studied here: (1) absolute abundance of iGDGTs is positively correlated with temperature and negatively correlated with pH; (2) two-way cluster analysis of polar iGDGTs indicates that individual samples cluster primarily according to temperature; (3) temperature is the strongest environmental correlate to the NMS model based on polar iGDGT composition; and (4) polar iGDGTs displayed unique relative abundance profiles along the temperature gradient, which allows identification of potential temperature optima for individual lipids in these spring systems. Oxygen, pH, nitrite, and nitrate also correlated with the NMS model; however, these analytes increase with decreasing temperature along geothermal outflow systems, and may or may not be direct controls on iGDGT production.

Observed increases in iGDGT cyclization with increasing temperature are consistent with physiological adjustments to temperature by thermophiles having wide temperature ranges; we propose, however, some iGDGTs may be putatively attributed to microbial lineages known to inhabit springs in particular temperature ranges. For example, the high relative abundance of polar iGDGT-0 in samples ≤70°C, giving way to a higher relative abundance of iGDGT-3 and iGDGT-4 at samples ≥55 and ≥60°C, respectively, is consistent with a shift from habitats with methanogenic Euryarchaeota at lower temperatures toward a higher relative abundance of Crenarchaeota (Thermoprotei) at higher temperatures. The relative abundance of crenarchaeol, in contrast, showed no direct temperature relationships but was common in springs ≤70°C, suggesting a wide distribution for AOA in Great Basin hot springs.

### Conflict of interest statement

The authors declare that the research was conducted in the absence of any commercial or financial relationships that could be construed as a potential conflict of interest.
