# Supplementary material for: The distribution and abundance of archaeal tetraether lipids in U.S. Great Basin hot springs
Source: Front Microbiol. 2013 Aug 28;4:247. doi: 10.3389/fmicb.2013.00247 (PMC3755460; doi:10.3389/fmicb.2013.00247)
Supplement: Supplementary file 4 [file Presentation1.PDF]

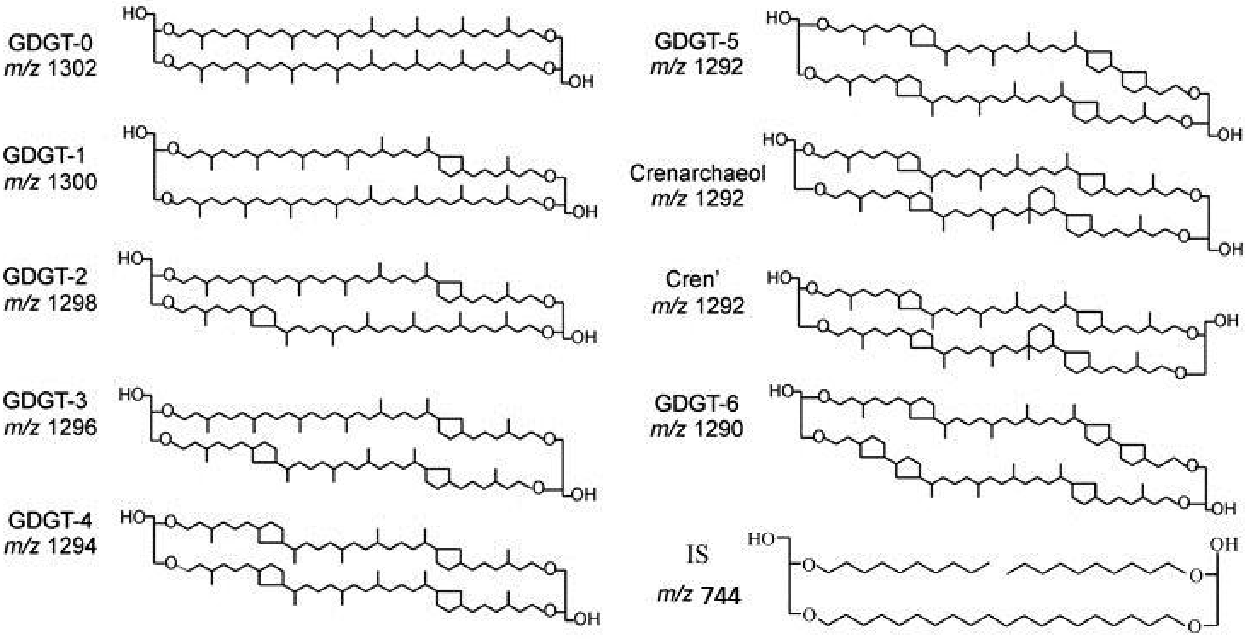

Figure S1: Archaeol core lipids found in Great Basin hot springs. Cren' is crenarchaeol isomer; IS is an internal standard.
