# Supplementary material for: The distribution and abundance of archaeal tetraether lipids in U.S. Great Basin hot springs
Source: Front Microbiol. 2013 Aug 28;4:247. doi: 10.3389/fmicb.2013.00247 (PMC3755460; doi:10.3389/fmicb.2013.00247)
Supplement: Supplementary file 5 [file Presentation2.PDF]

Great Boiling Spring GBS Sites A - C

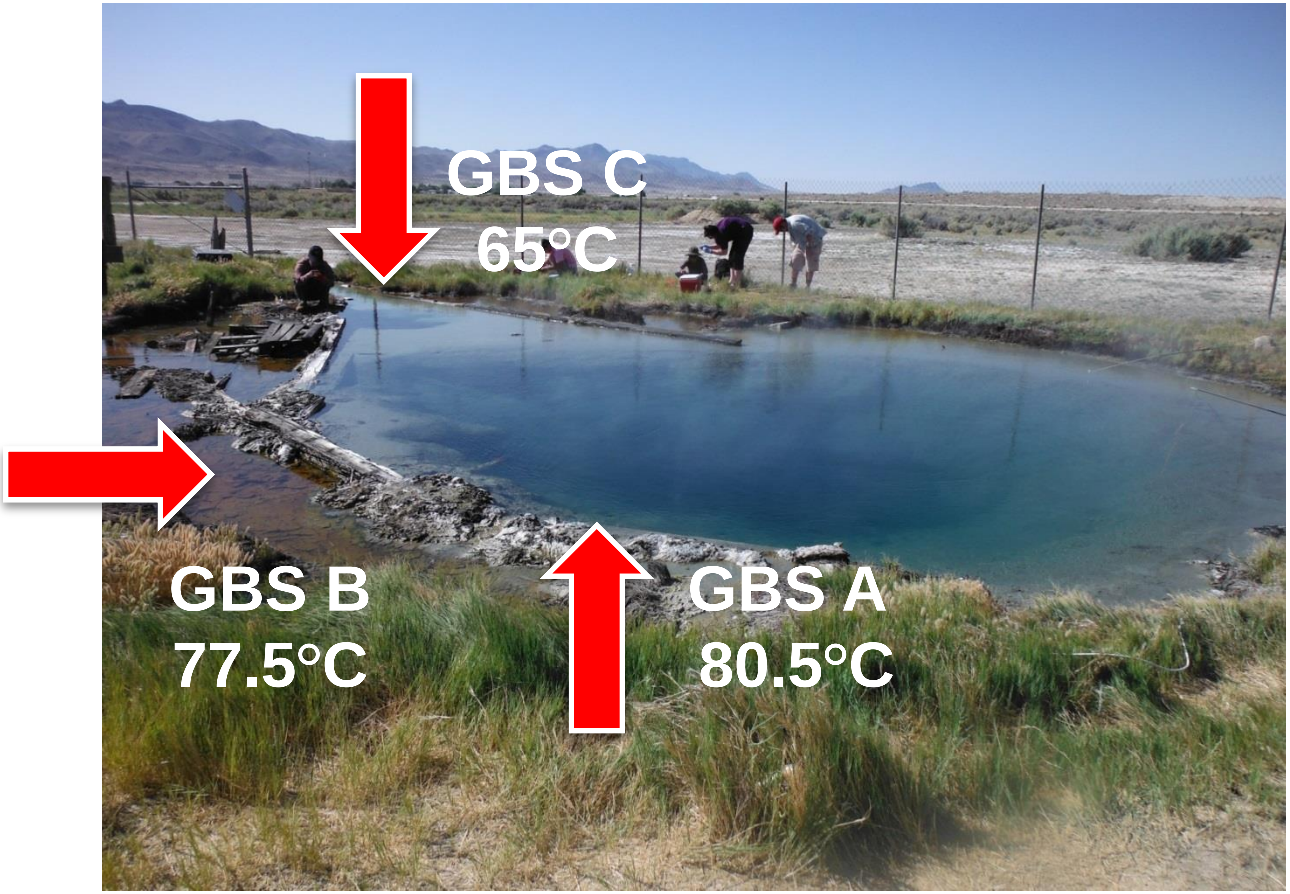

GBS A - Sediments homogenous, site near stick (pH = 7.41)

GBS B - Sediments homogenous, site near pallet (pH = 7.35)

GBS C - Sediments homogenous, near GBS outflow and cellulose enrichments (pH = 7.69)

Great Boiling Spring
GBS 61 (Y) - 61ºC

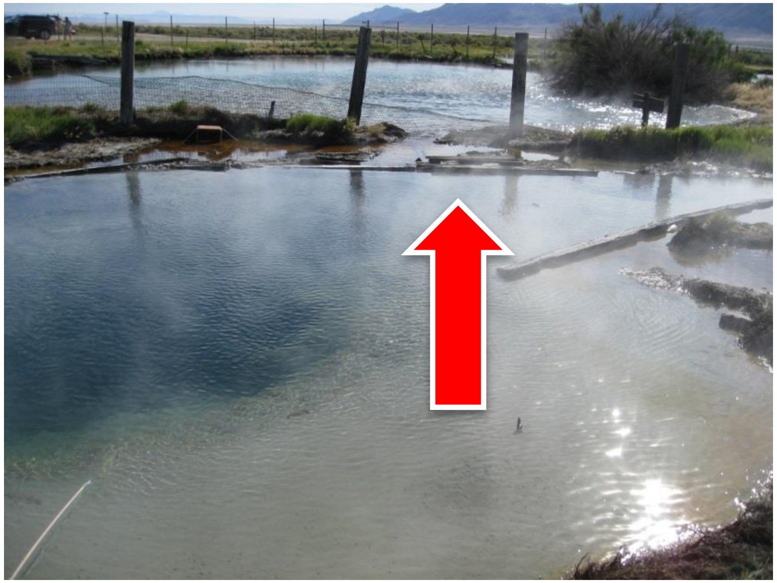

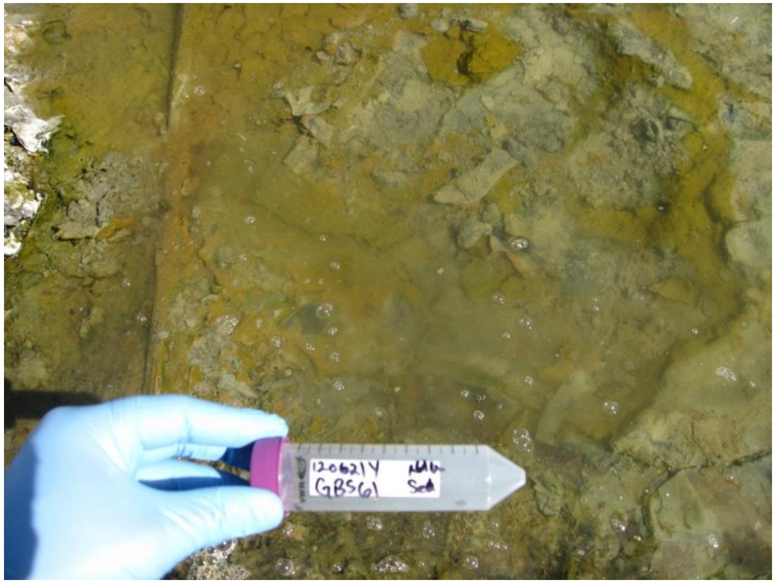

GBS 61 (Y) - Gray/Black, gritty sediment with orange microbial mat in outflow north of spring (pH = 8.00)

Sandy's Springs West
SSW Source 80ºC

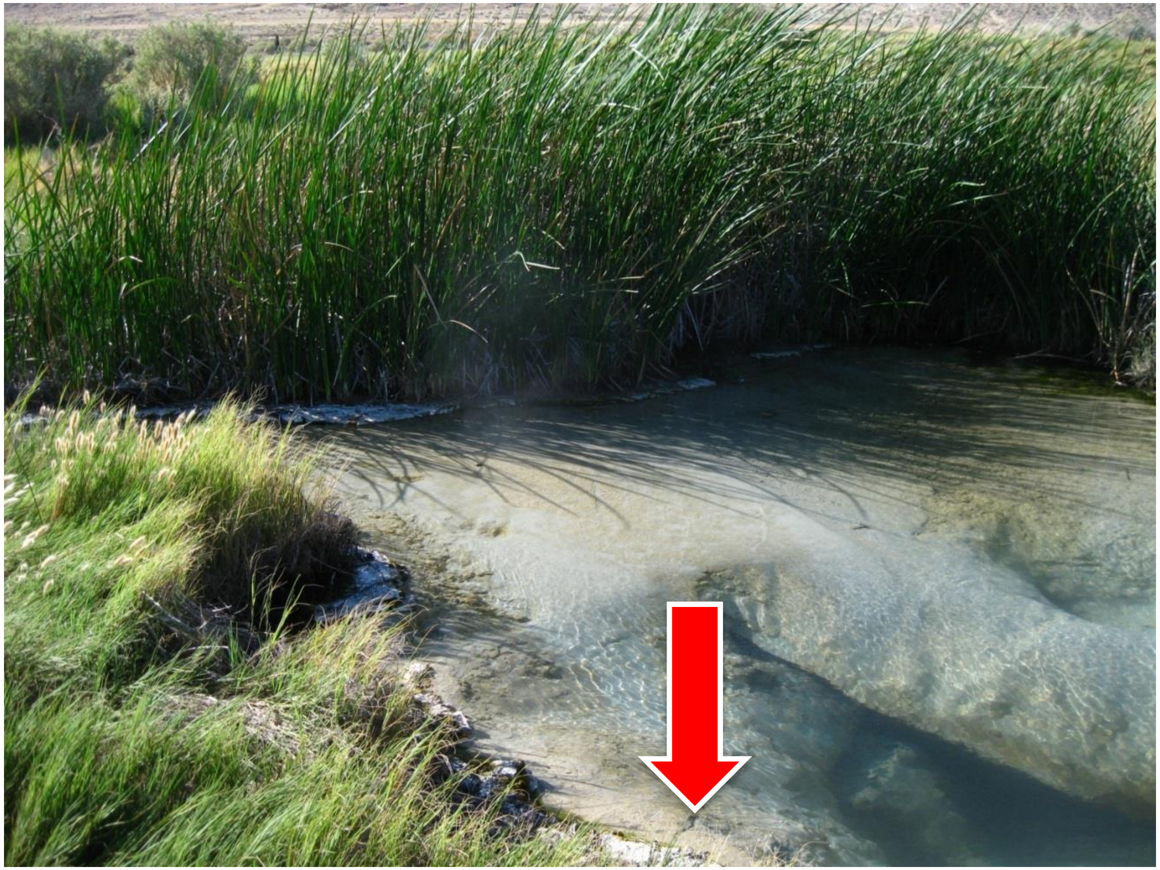

SSW Source - Sediments homogenous (pH = 7.37)

Sandy's Springs West
SSW 70 - 70ºC

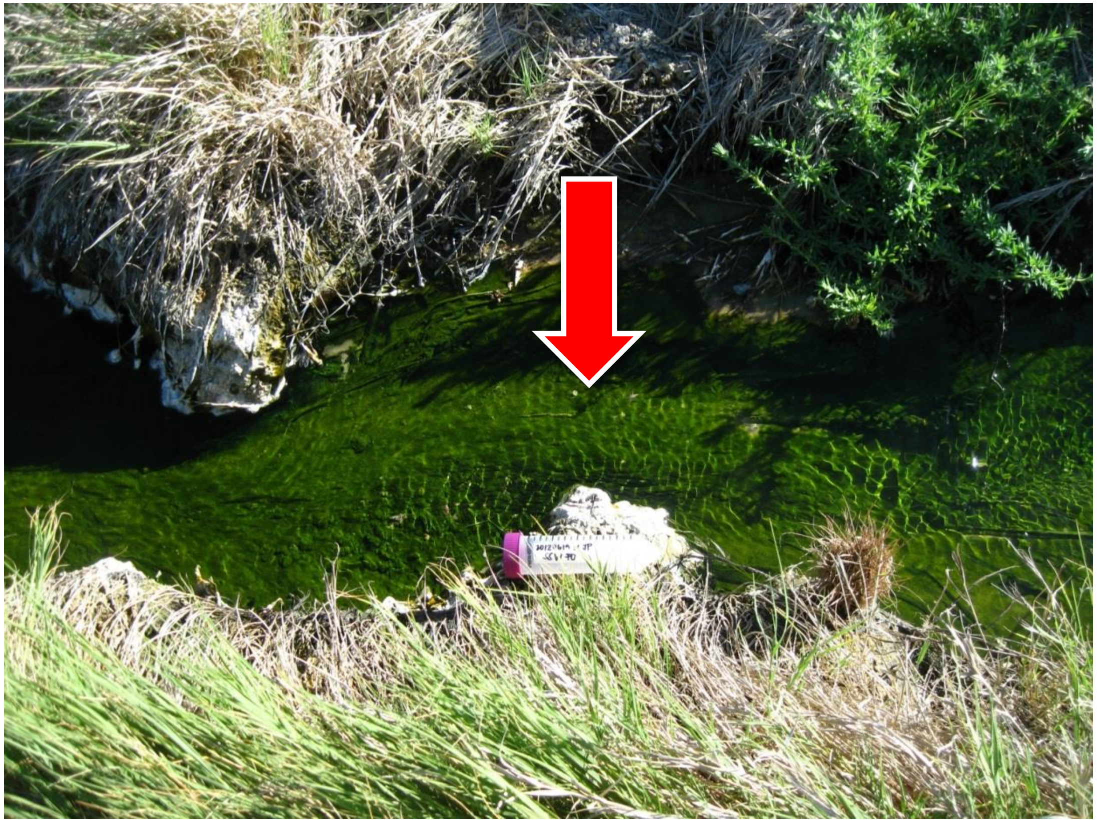

SSW 70 - Brilliant green mat with streamers with white precipitate on side (pH = 7.86)

Sandy's Springs West
SSW 60 – 59.5ºC

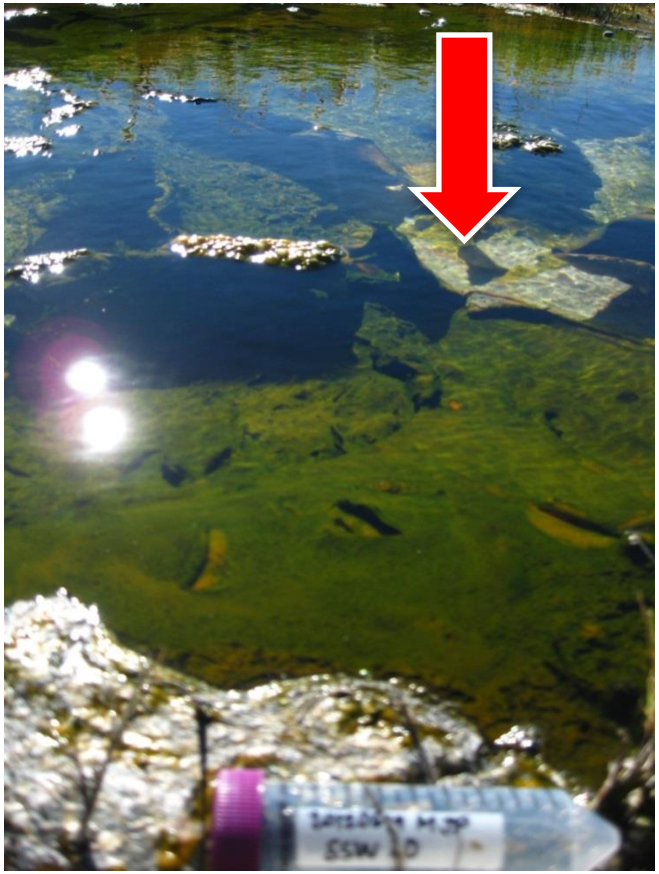

SSW 60 - Brilliant laminated microbial mat green above, orange below, peeling up at places near outflow SSE (pH 7.90)

Sandy's Springs West
SSW OF – 50.5ºC

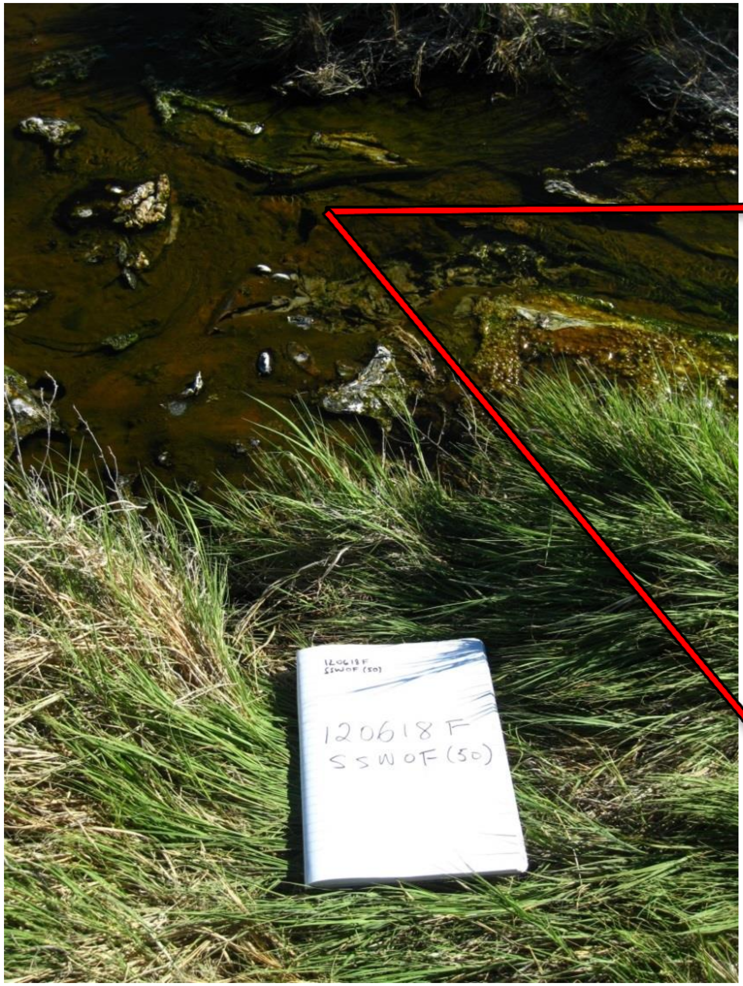

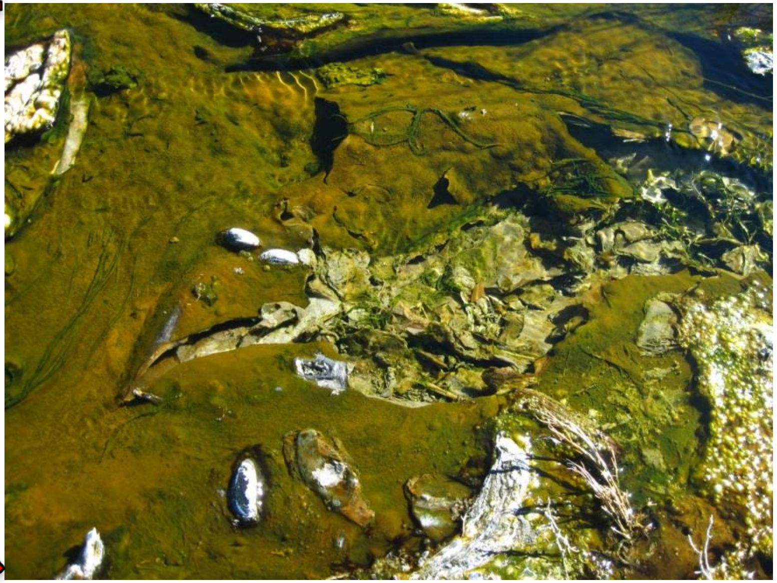

SSW OF - Laminated microbial mat, dark green surface layer, rusty lower layer, small amount sediment sampled (pH = 8.17)

Sandy's Springs West
SSW 40 – 39.4ºC

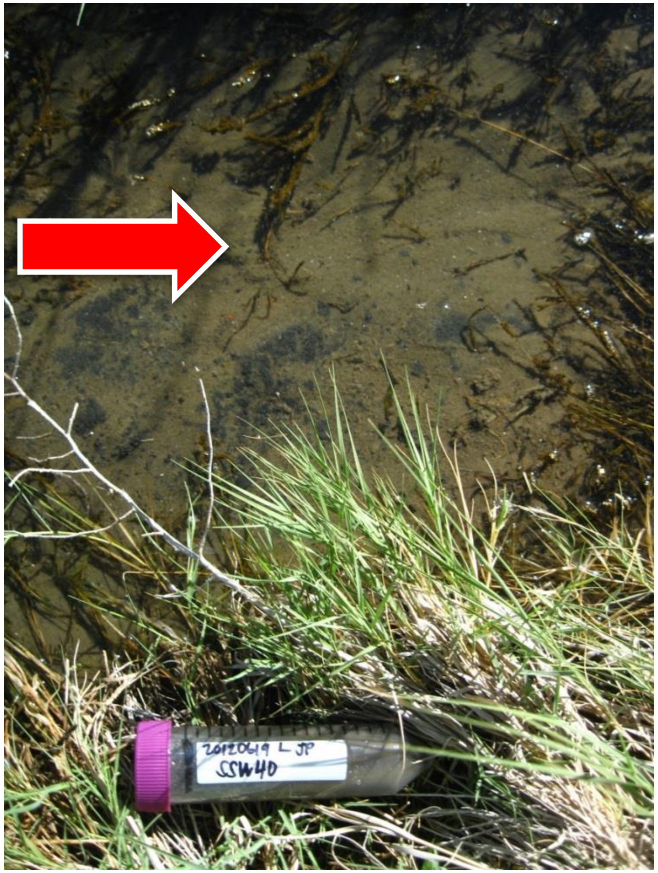

SSW 40 - Dark, brown, fluffy sediment with $CaCO_3$-covered roots; roots were avoided (pH = 8.40)

Rick’s Hot Creek
RHC 5 – 90.2ºC

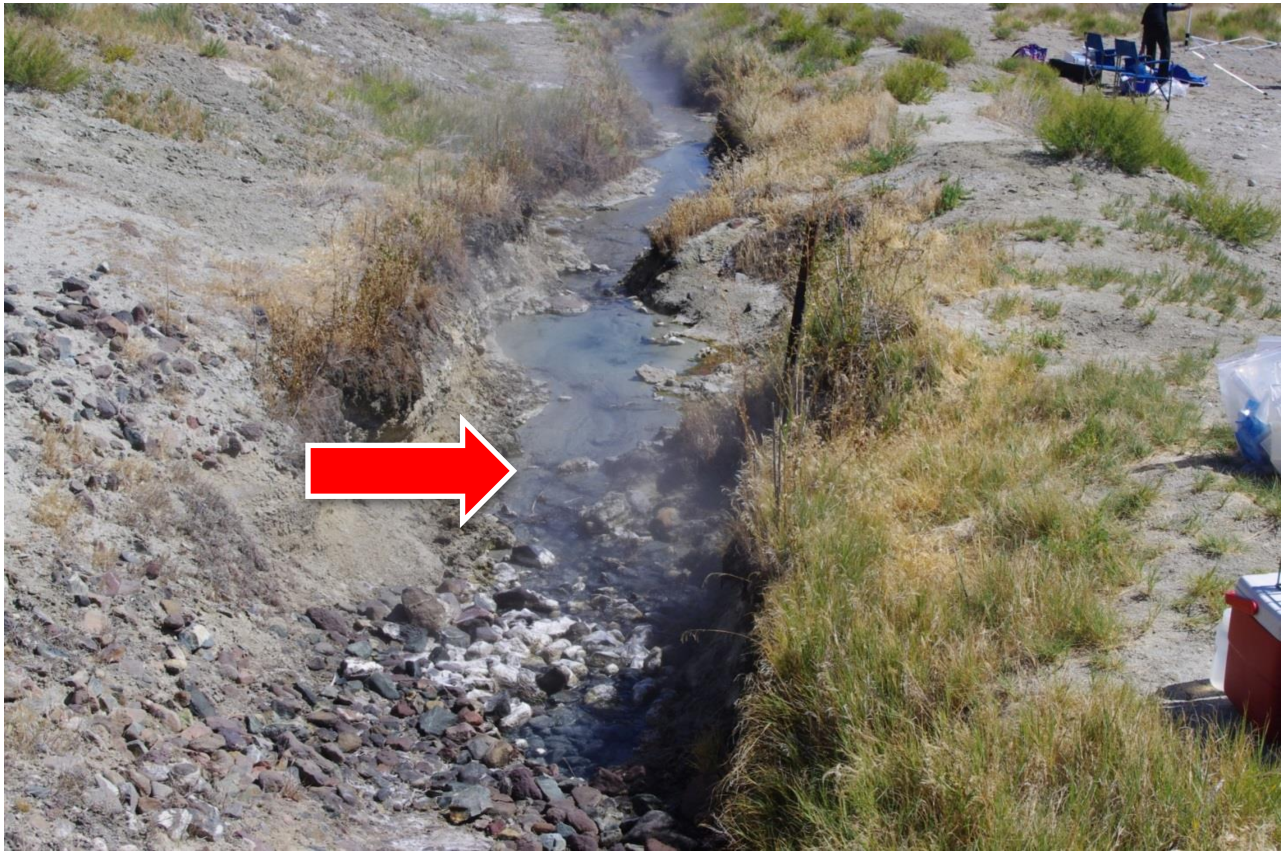

RHC 5 - Fine sediment/clay, gray (pH = 7.49)

Fly Geyser
FG 60 – 60ºC

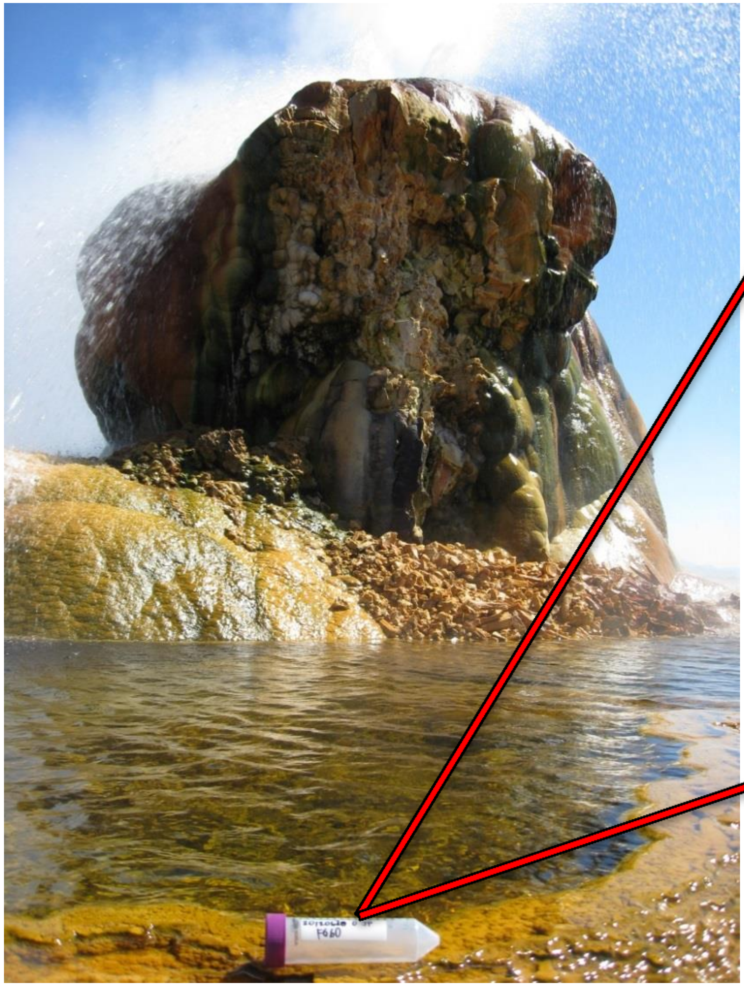

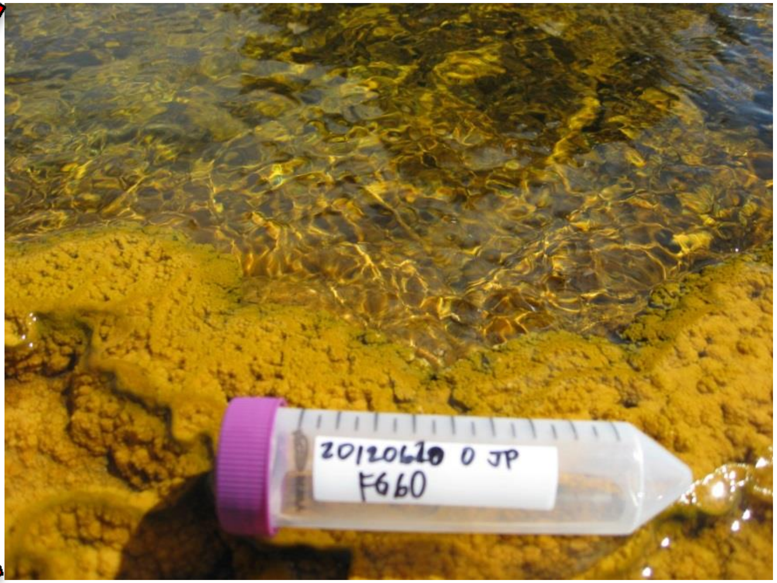

FG 60 - Scalloped orange microbial mat from top of terrace/source pool, gray, and brown substrate below, hint of sulfur, coarse material, difficult to homogenize (pH = 8.37)

Fly Geyser
FG 50 - 50ºC

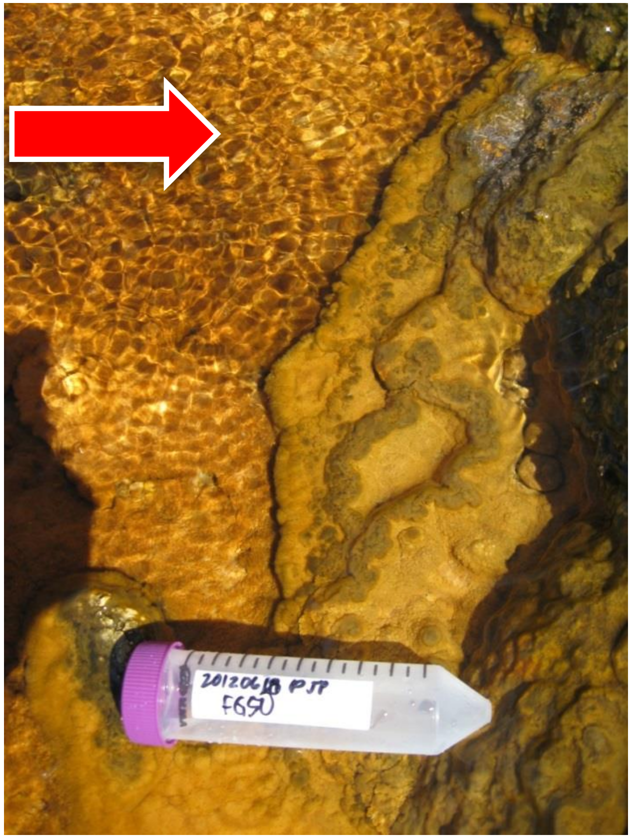

FG 50 - Red/Orange microbial mat in pool below major terrace with fine gray sediment below (pH = 8.60)

Fly Geyser
FG 42 - 42ºC

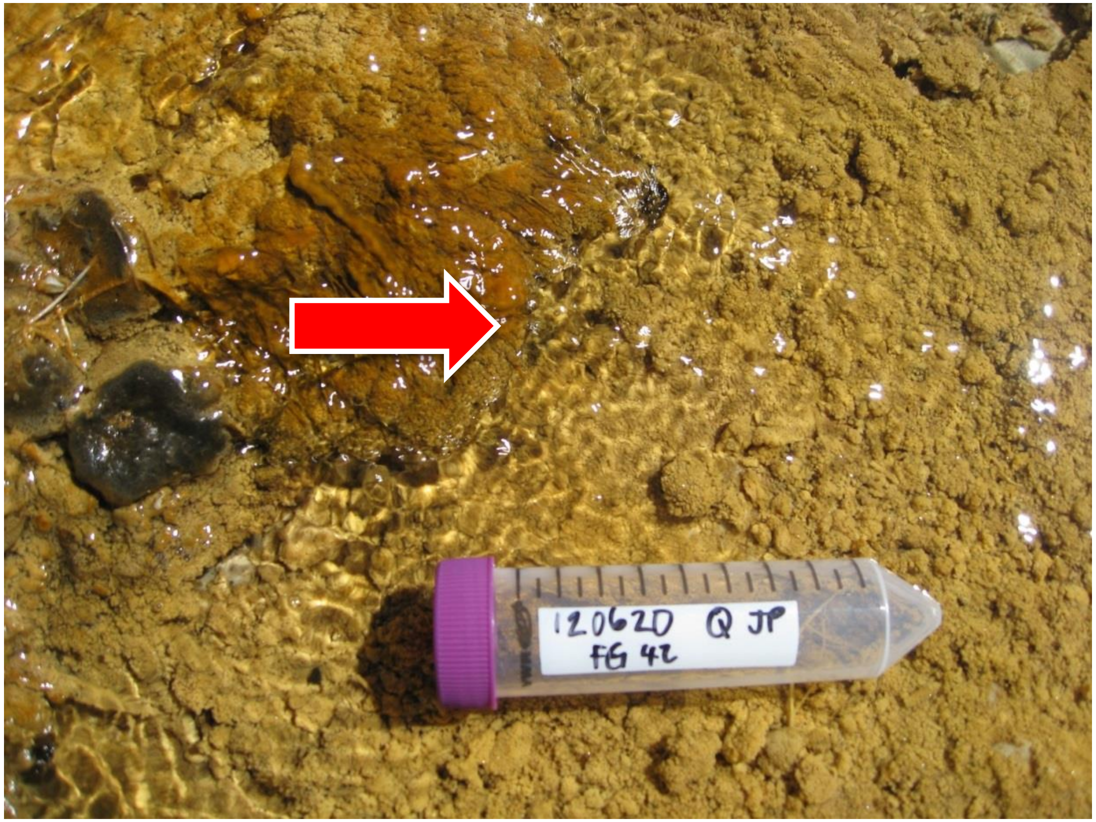

FG 40 - Red/Orange microbial mat in very bottom of terrace structure, difficult to homogenize (pH = 8.80)

Double Hot Springs DH Source – 79.6ºC

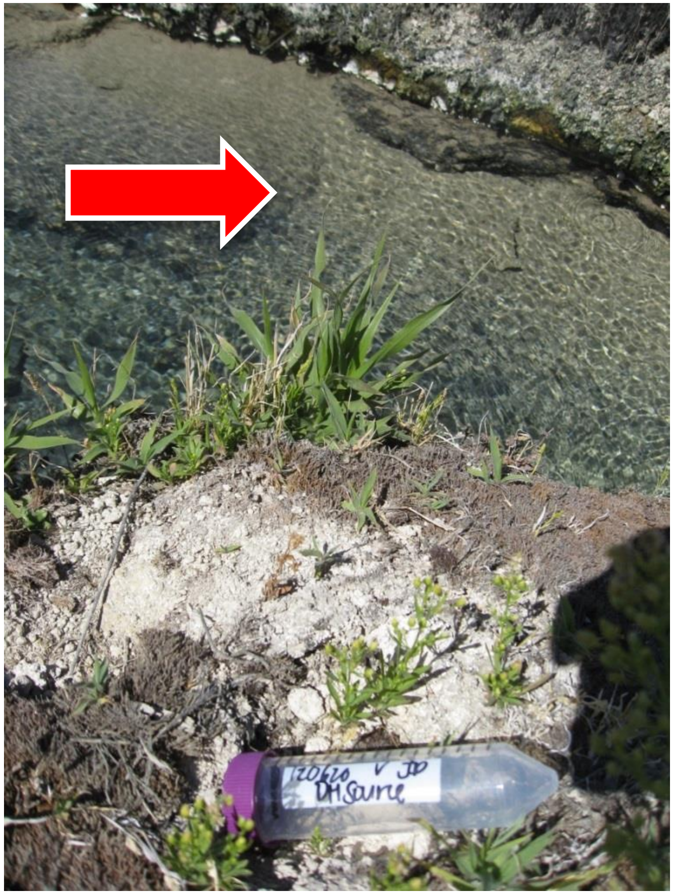

DH Source – Fine, fluffy gray clay above with material difficult to homogenize (pH = 8.05)

Double Hot Springs
DH 70 – 69.6ºC

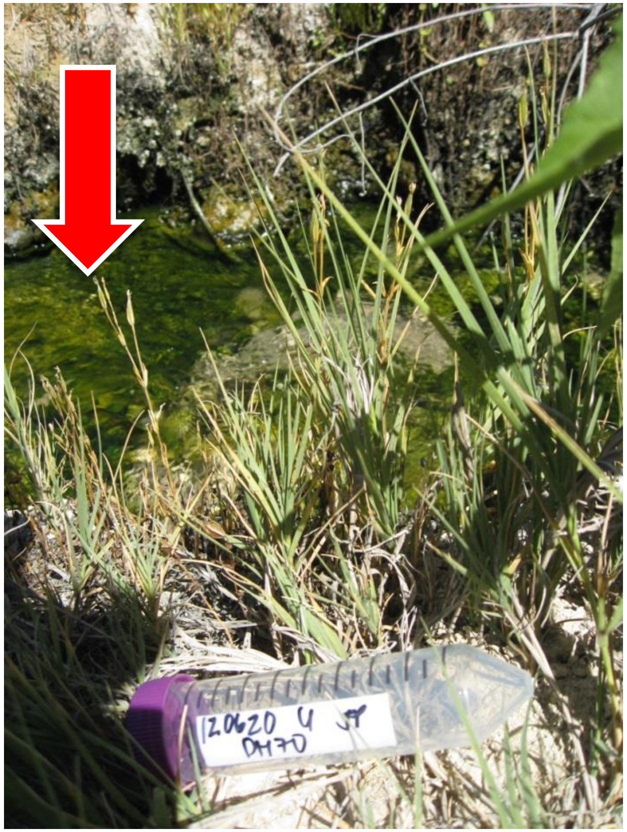

DH 70 - Green encrusted, tough microbial mat, difficult to homogenize (pH = 8.37)

Double Hot Springs
DH 60 – 59.9ºC

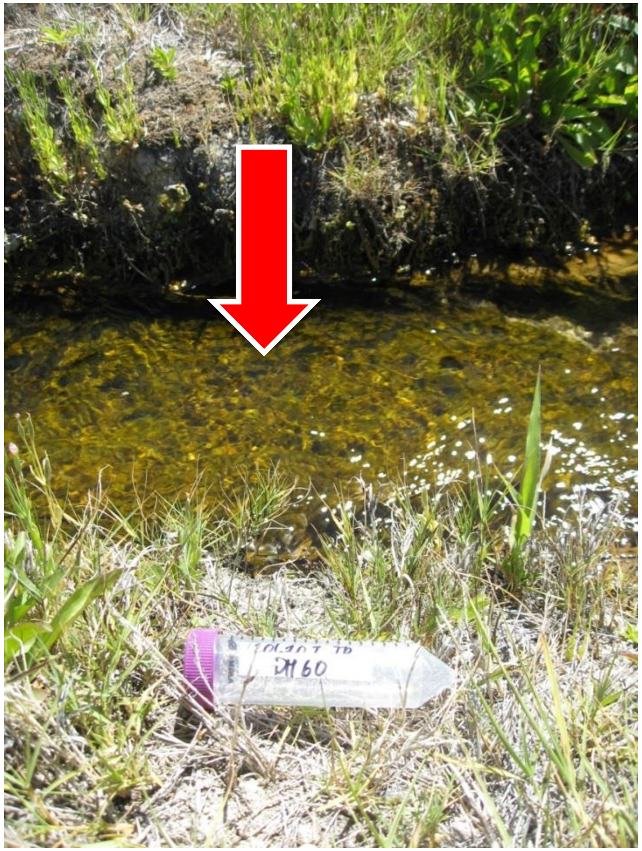

DH 60 - Brilliant gold/green hair-like streamers, very thick growth, easy to homogenize (pH 8.74)

Double Hot Springs DH Source 2 – 54.5ºC

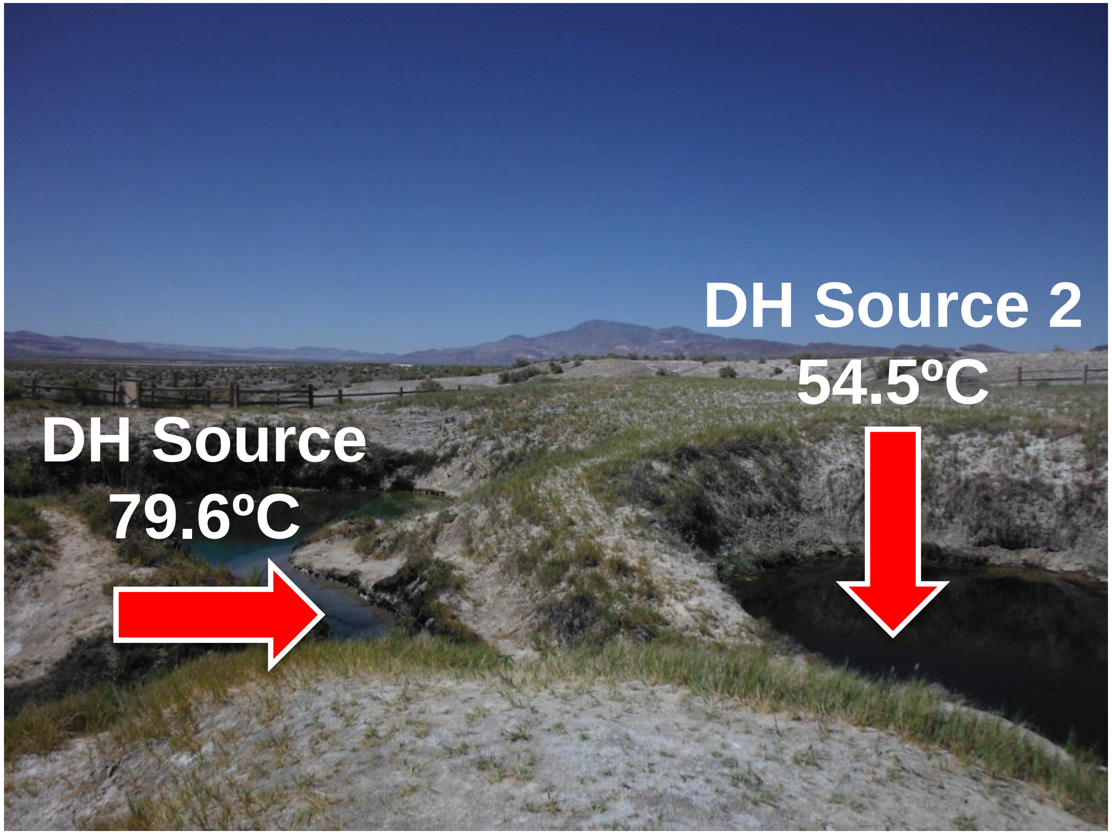

DH Source 2 = Soft black sediments (pH = 8.50)

Double Hot Springs
DH 50 – 49.9ºC

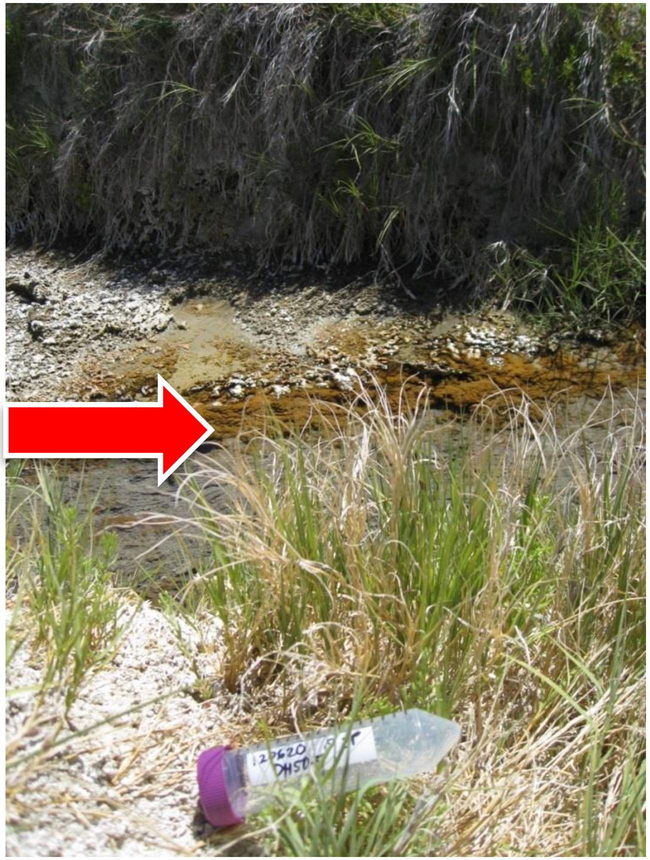

DH 50 - Thin microbial mat above with black/gray sediment below, stinky (pH = 9.09)

Double Hot Springs
DH 43 – 43.5ºC

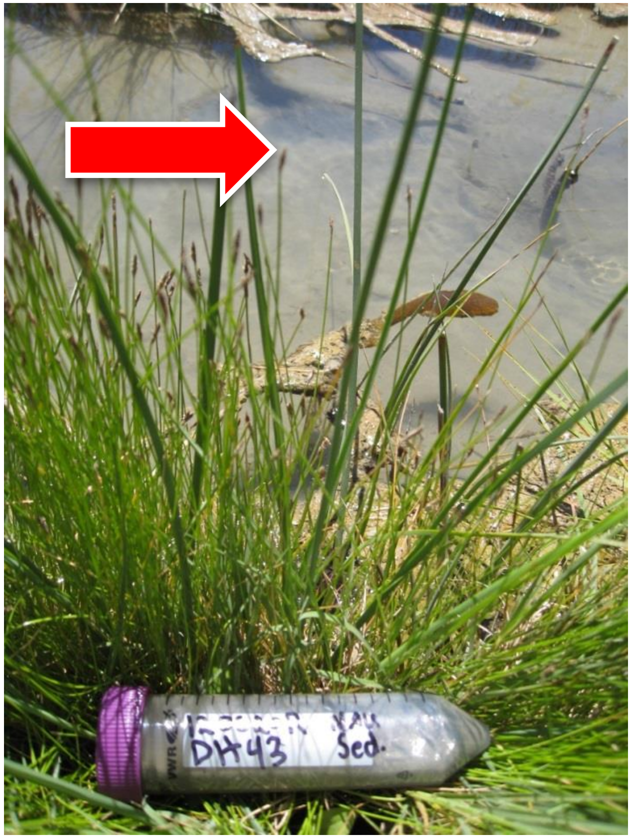

DH 43 - Sparse growth of orange microbial mat in gray clay (pH = 9.24)

Double Hot Springs
DH 31 - 31ºC

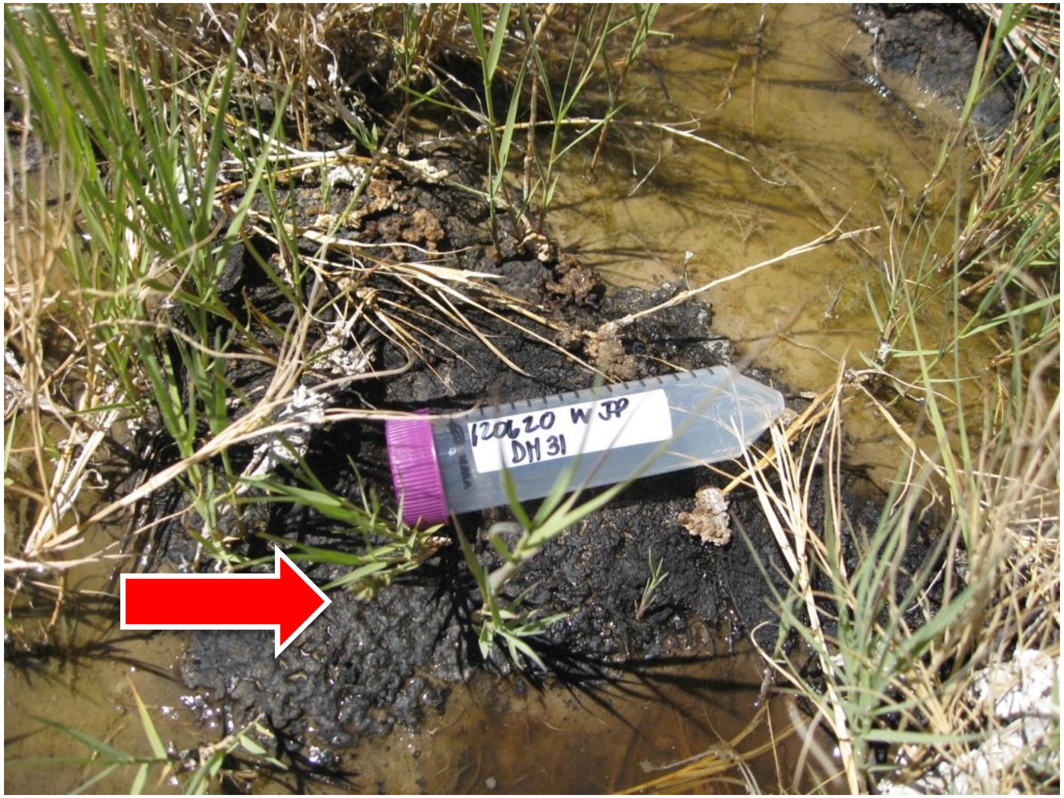

DH 31 - Black soil associated with tufts of grass in shallow swamp in alkali soil, viscous mixture (pH = 10.70)

Black Rock hot spring
BR 45 – 45.5ºC

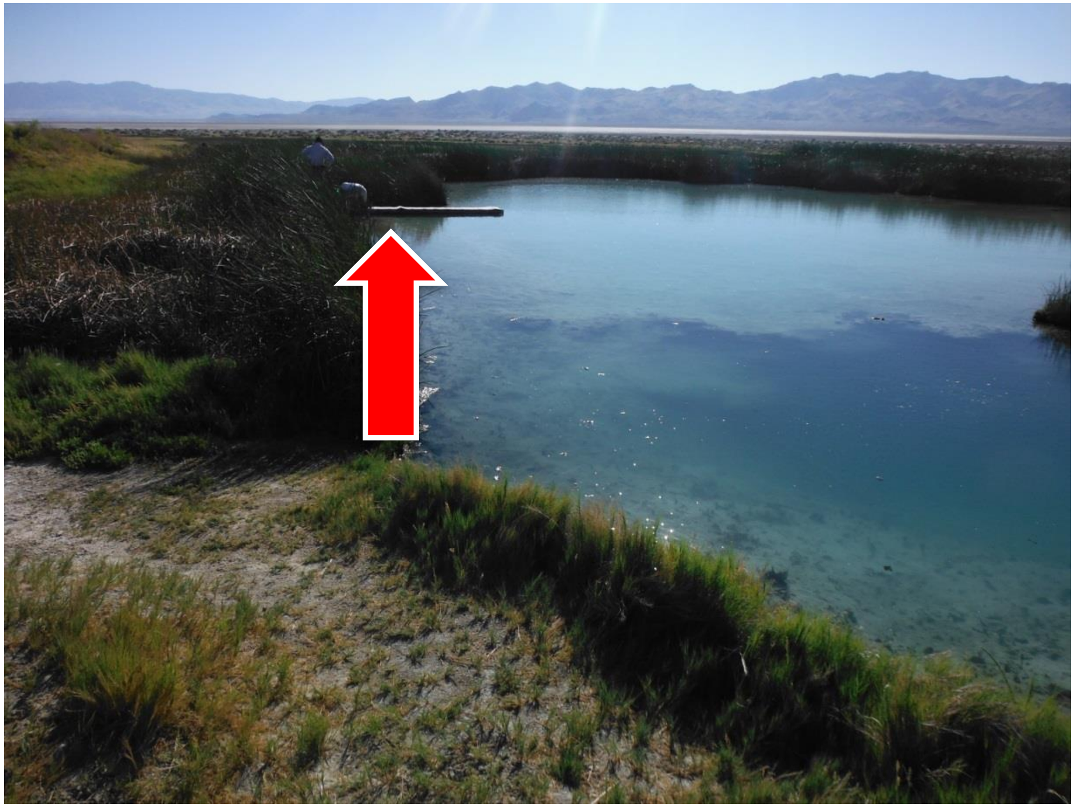

BR 45 - Fine gray sediments (pH 7.88)

Eagleville hot spring
EV 44 & EV 43 – 43.8ºC

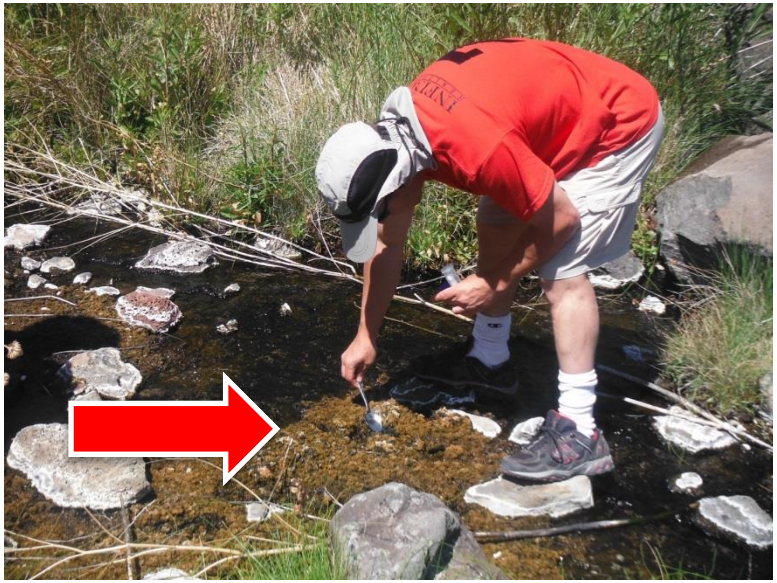

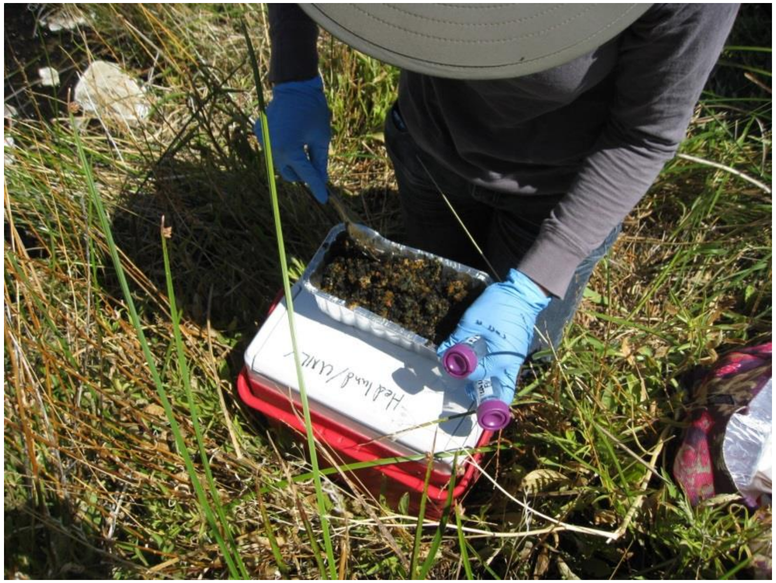

EV 43 - Orange coral-like microbial mat above waterline with visible layers below of green, black, and clear with sand below (sample included orange, green, and black layers) (pH = 9.73)

EV 44 - Same as above but clear layer below sampled (pH = 9.73)

Surprise Valley
SV Source – 86ºC

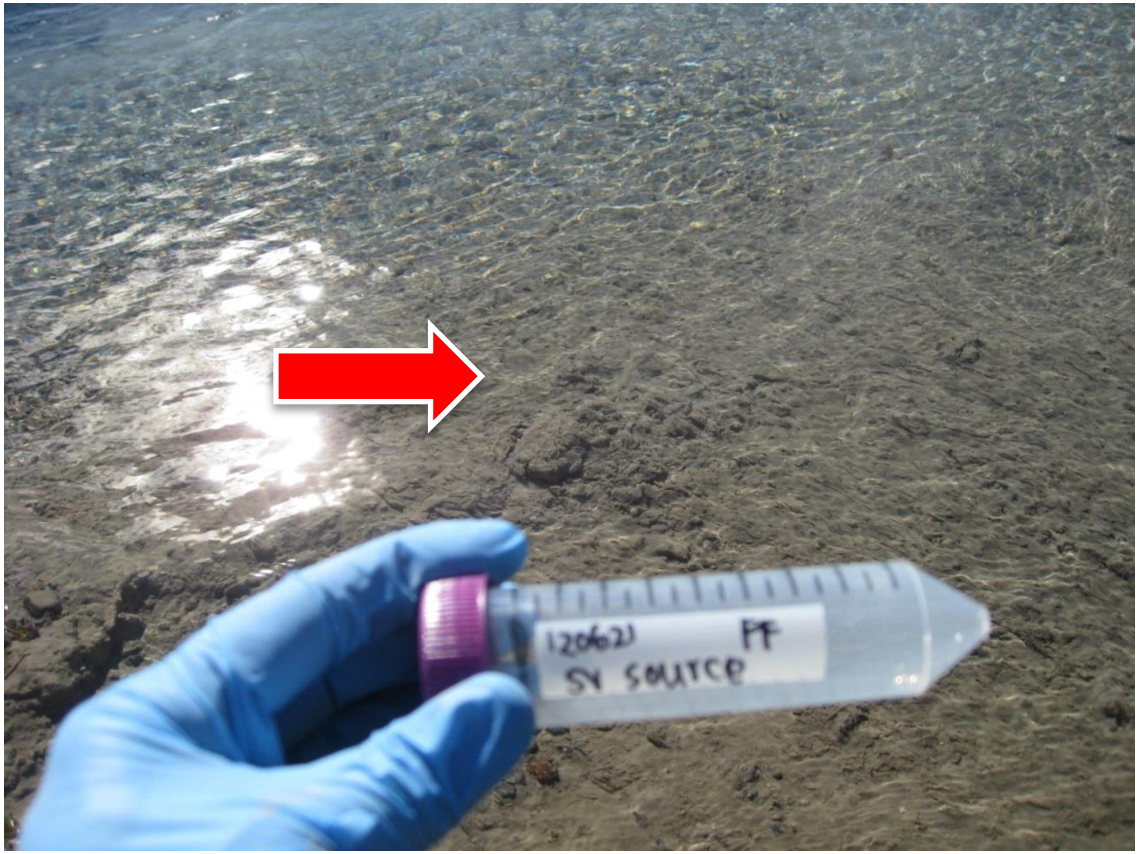

SV Source - Fine clay sediment, easy to homogenize, source pool also had areas with large gravel, clear microbial mat visible (pH = 8.34)

Surprise Valley
SV 70 – 69.2ºC

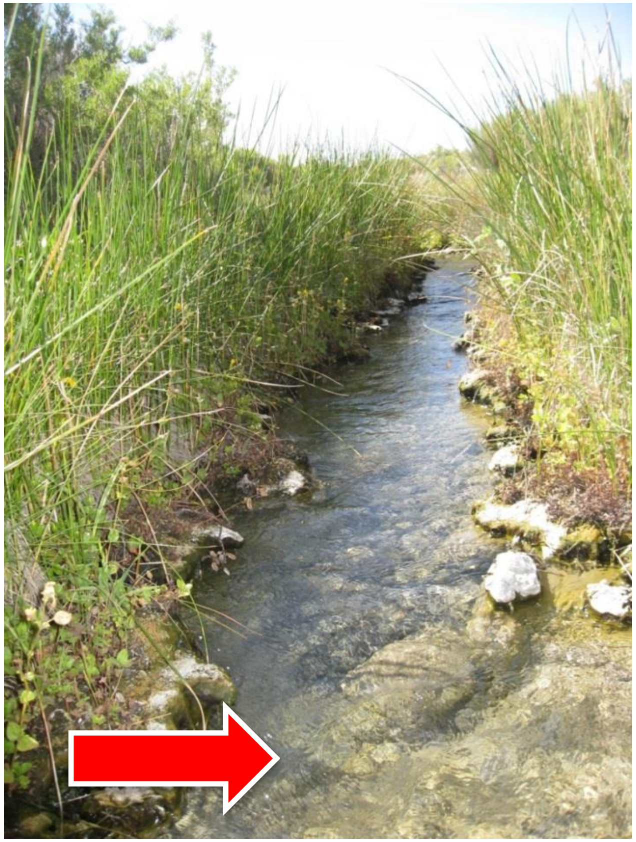

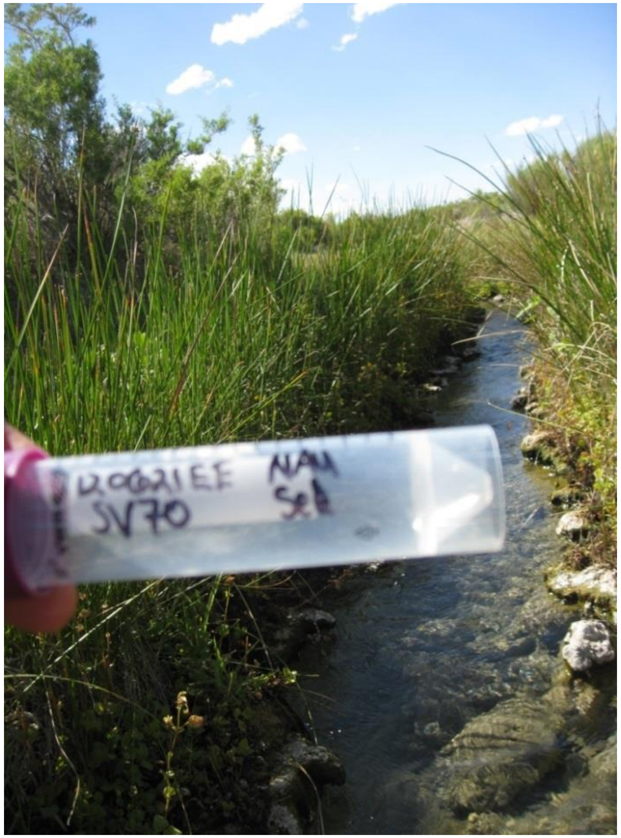

SV 70 - Clay-like bottom with sparse microbial growth; small photosynthetic fringe on the sides (pH = 8.50)

Surprise Valley
SV 60 – 59.6ºC

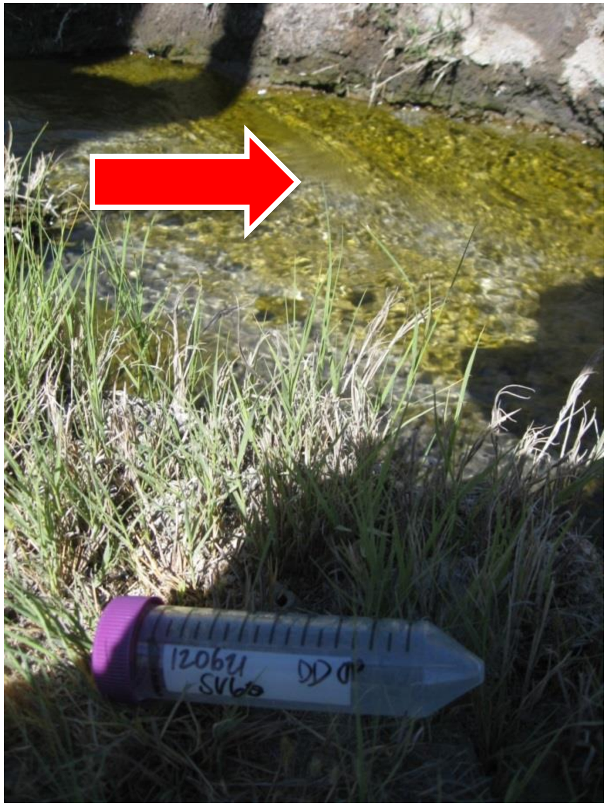

SV 60 - Green on sides and gray in the middle, sparse growth (pH = 8.64)

Surprise Valley
SV 50 – 48.5^{o}C

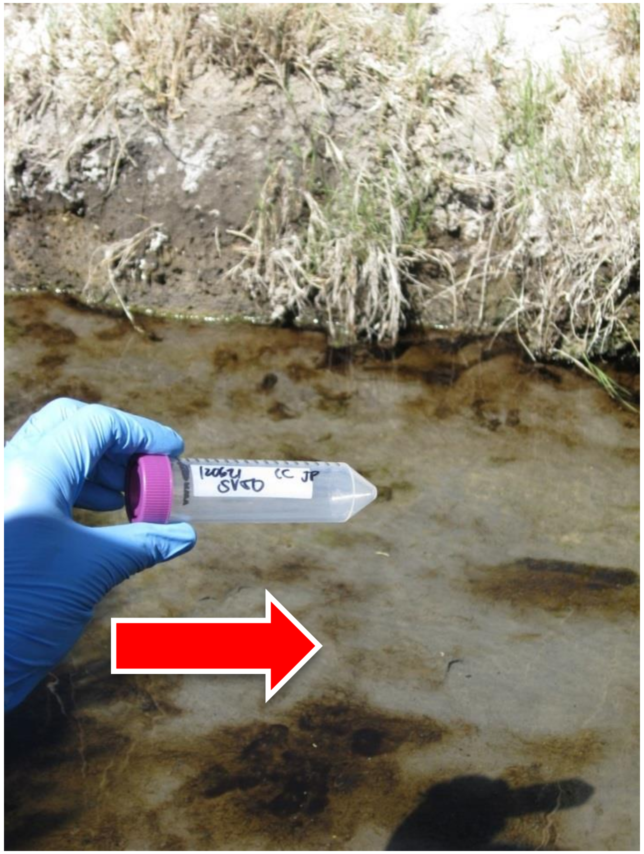

SV 50 - Brown/Gray sediment with sparse, green microbial mat above (pH = 8.78)

Surprise Valley
SV 40 – 41.3ºC

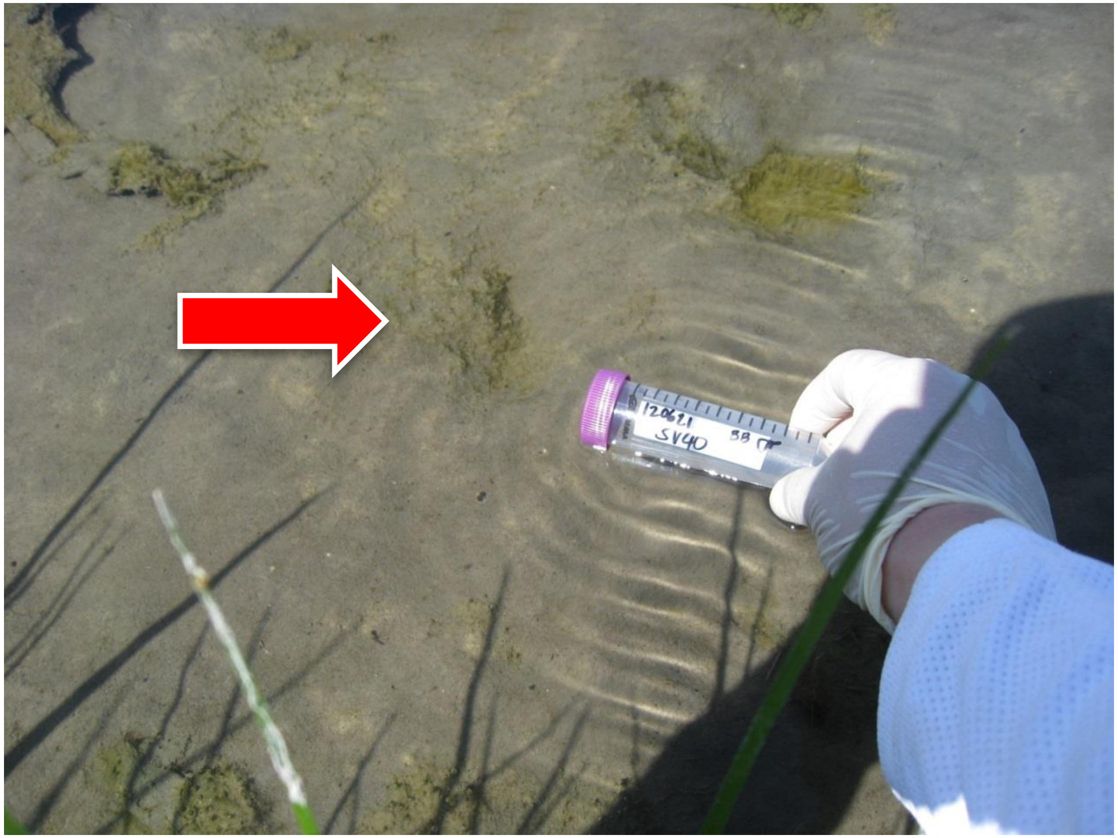

SV 40 - Green floating microbial mat, difficult to homogenize, swampy area, mat was patchy, covering ~70% of stream (pH = 9.07)

Surprise Valley hot spring SVX Source – 83.7ºC

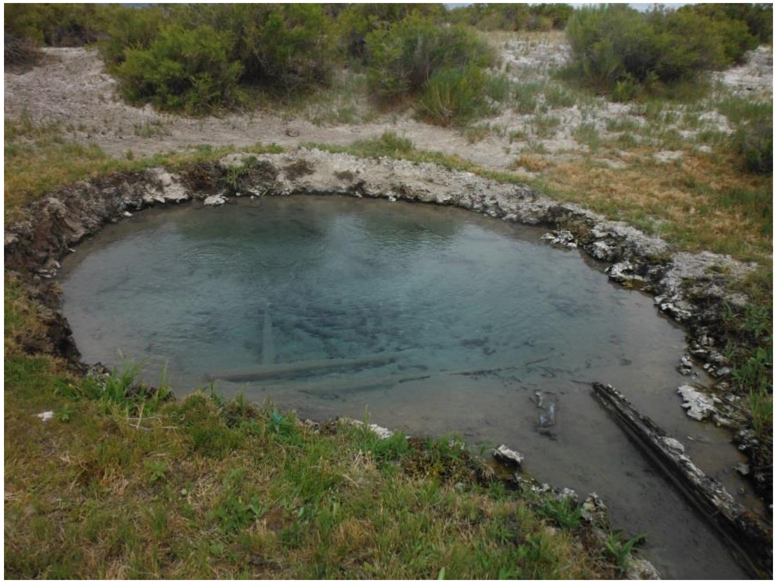

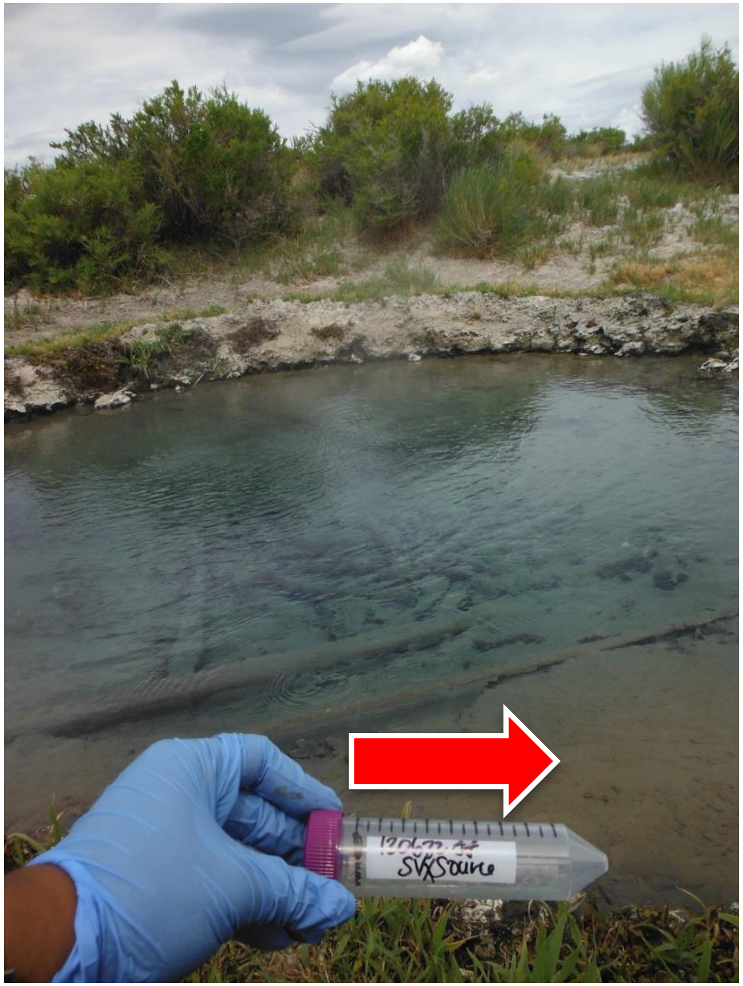

SVX Source - Soft gray sediment (pH = 8.41)

Surprise Valley hot spring SVX 70 – 68.5ºC

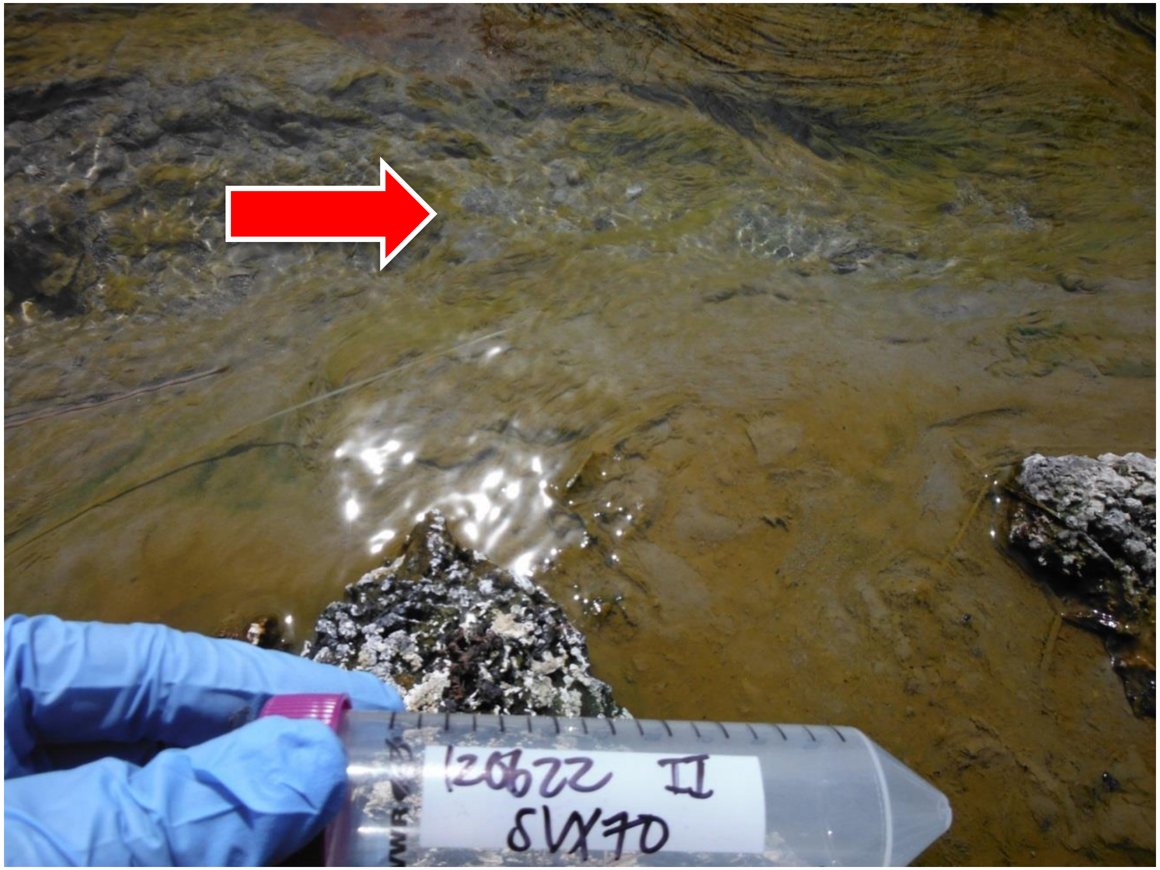

SVX 70 - Thick orange/green microbial mat on side of stream with gray middle, silky, fine gray sediments (pH = 8.58)

Surprise Valley hot spring SVX 60 – 60.6ºC

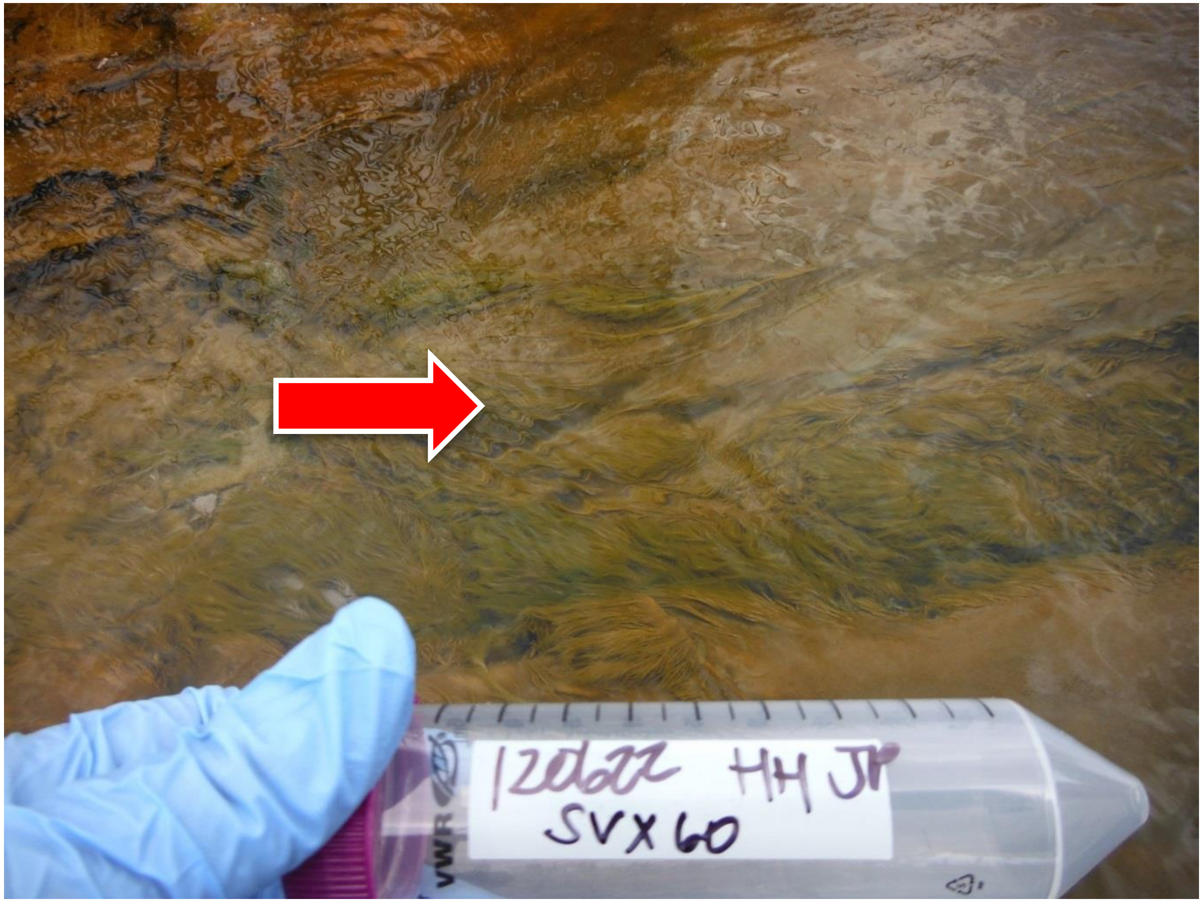

SVX 60 - Thick orange/green microbial mat on surface with fine gray sediments, streamers also sampled (pH = 8.72)

Surprise Valley hot spring SVX 50 - 50ºC

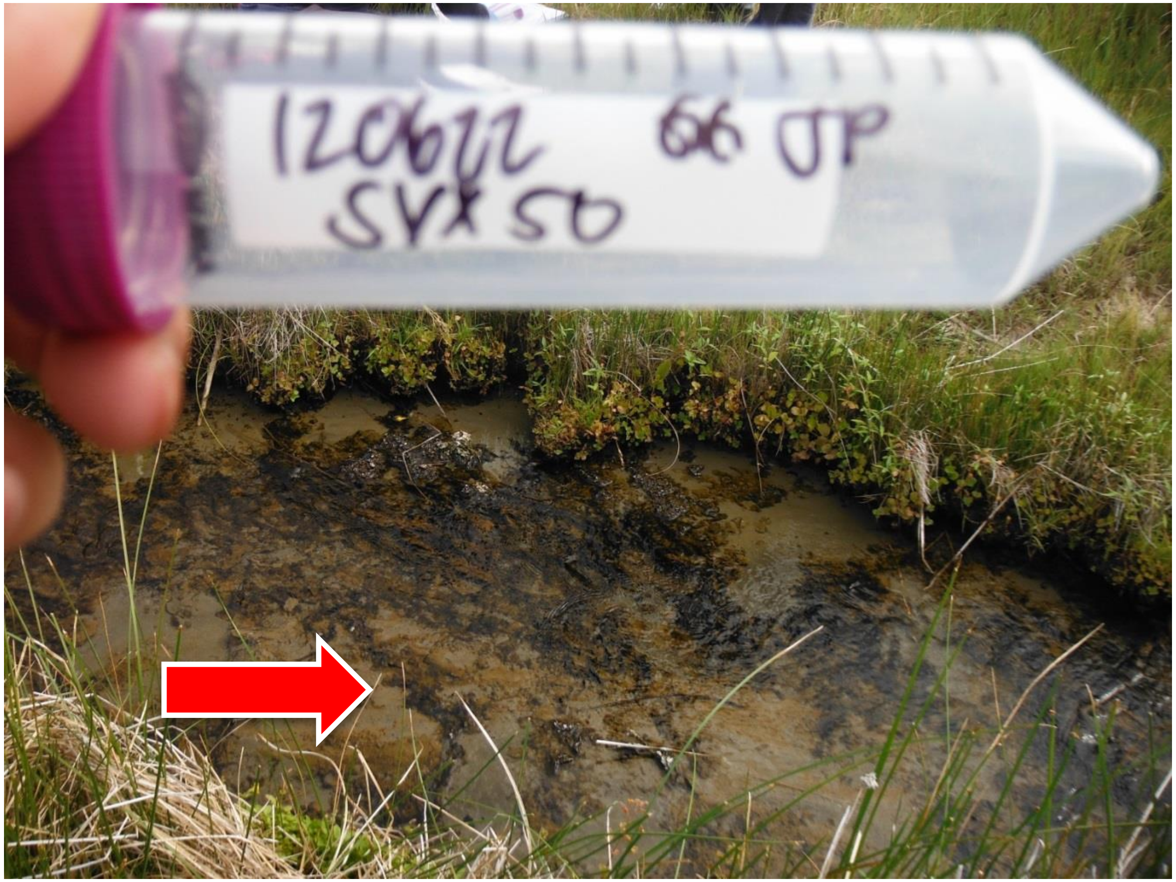

SVX 50 - Black/Orange microbial mat on surface with fine gray sediments below (pH = 8.98)

Surprise Valley hot spring SVX 2 – 83.4ºC

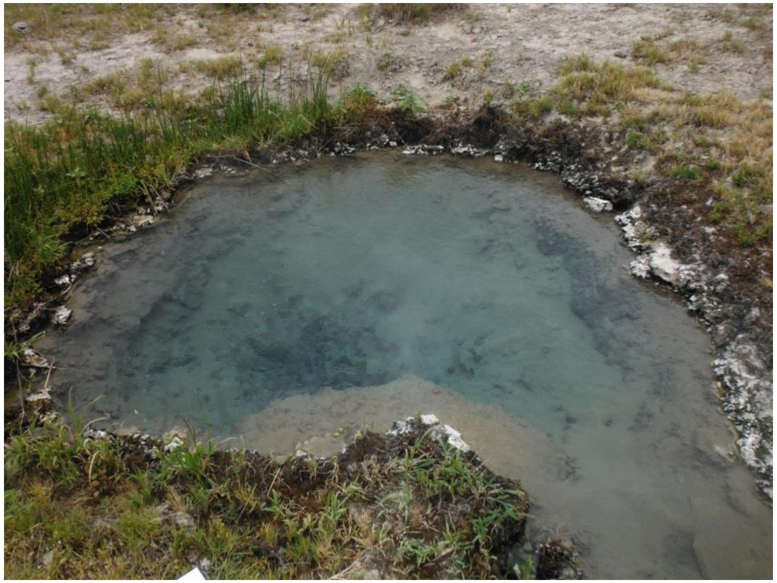

SVX 2 - Soft gray sediment (pH = 8.24)

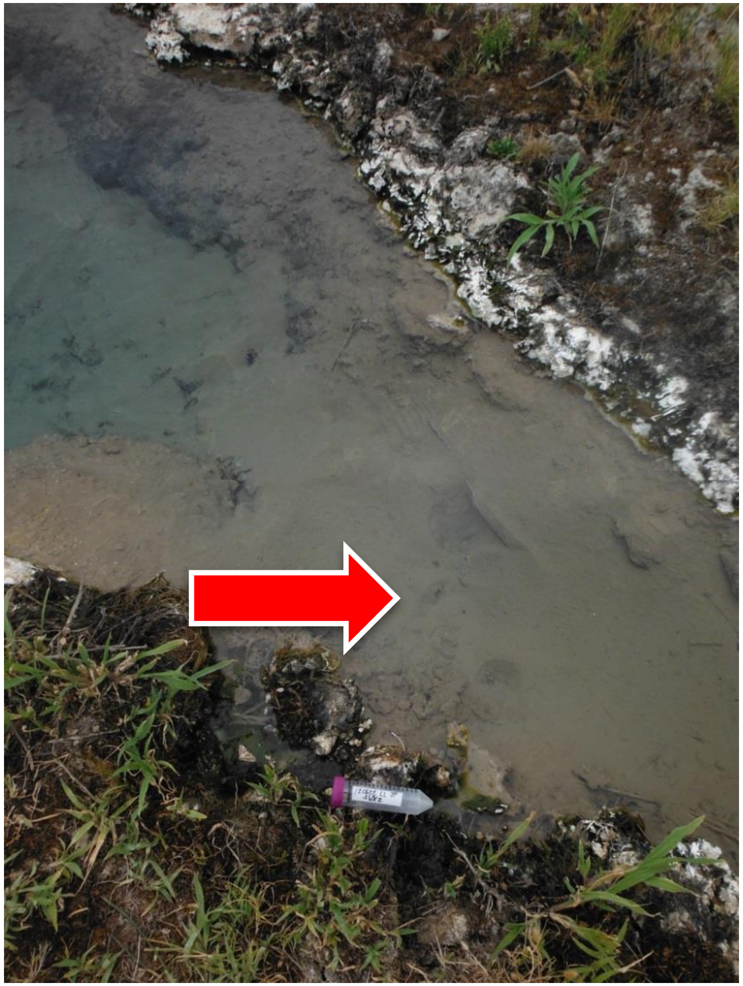

Surprise Valley hot spring SVX 1 – 76.5ºC

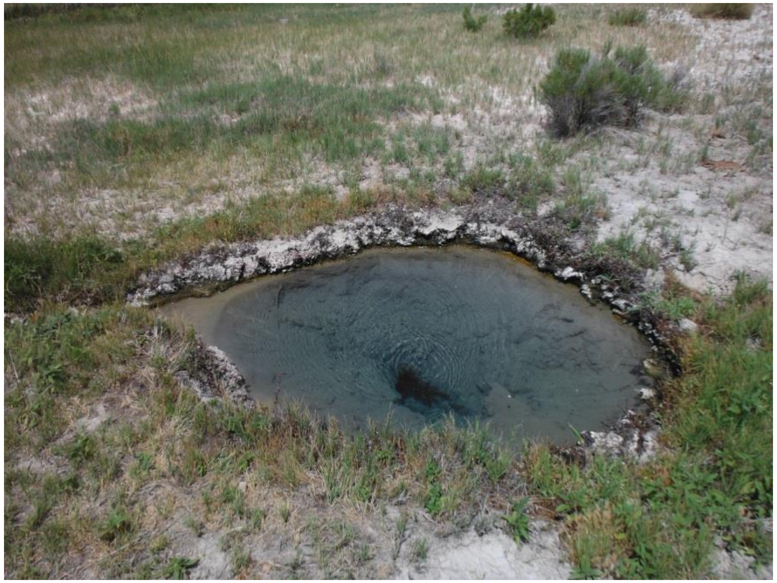

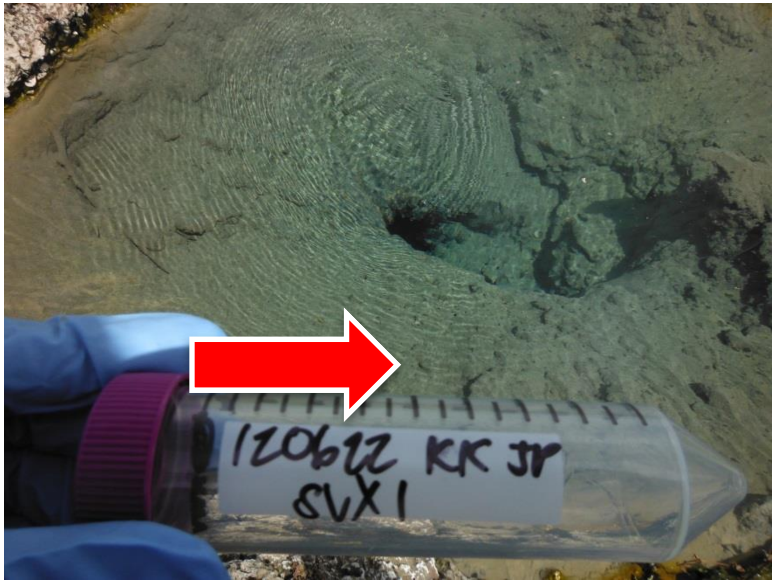

SVX 1 - Soft gray sediment (pH = 8.32)

Surprise Valley hot spring SVX 3 – 40.8ºC

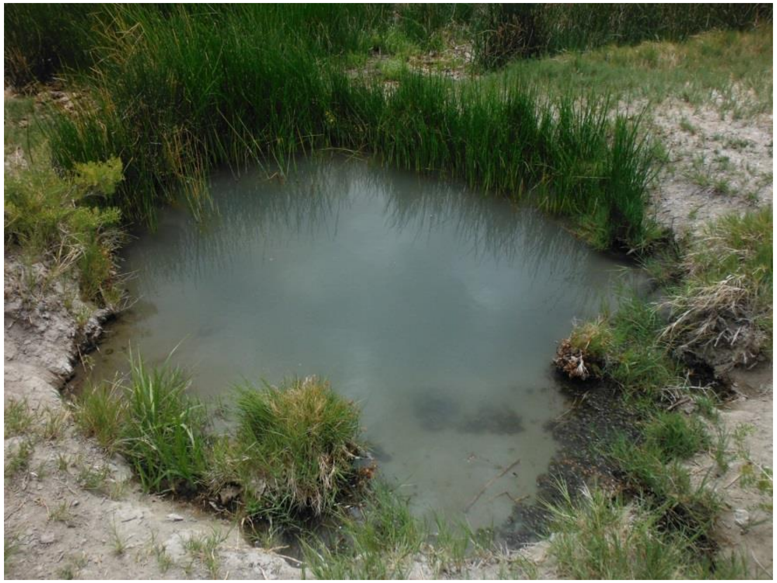

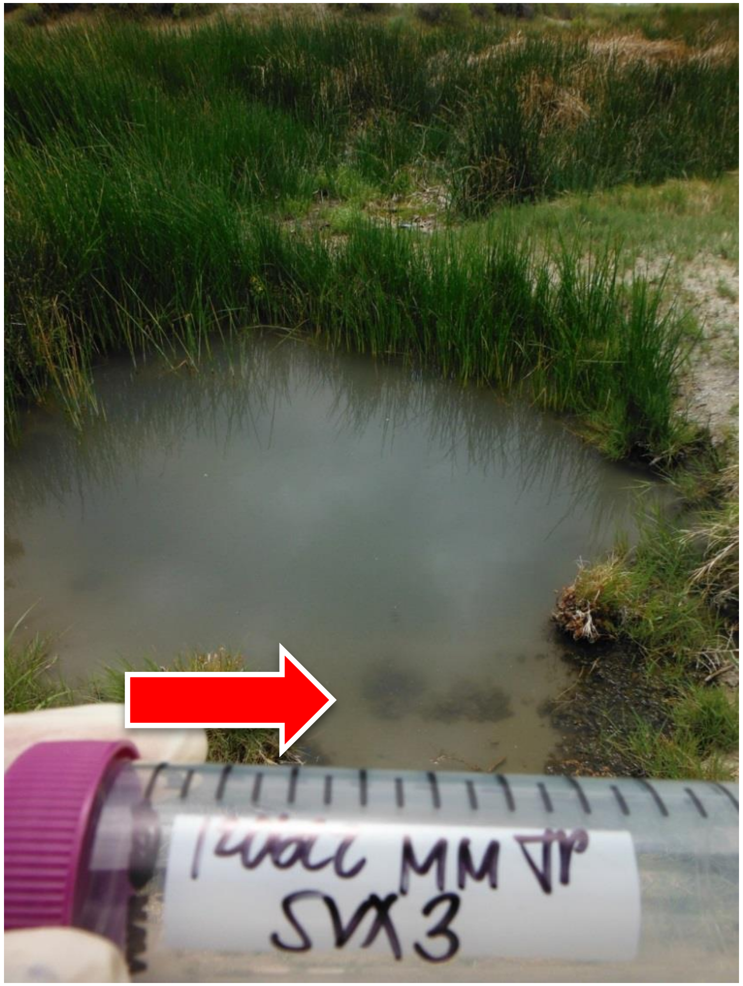

SVX 3 - Soft gray sediment with opaque water, several insects present (pH = 8.22)
