# Supplementary material for: The distribution and abundance of archaeal tetraether lipids in U.S. Great Basin hot springs
Source: Front Microbiol. 2013 Aug 28;4:247. doi: 10.3389/fmicb.2013.00247 (PMC3755460; doi:10.3389/fmicb.2013.00247)
Supplement: Supplementary file 6 [file Presentation3.PDF]

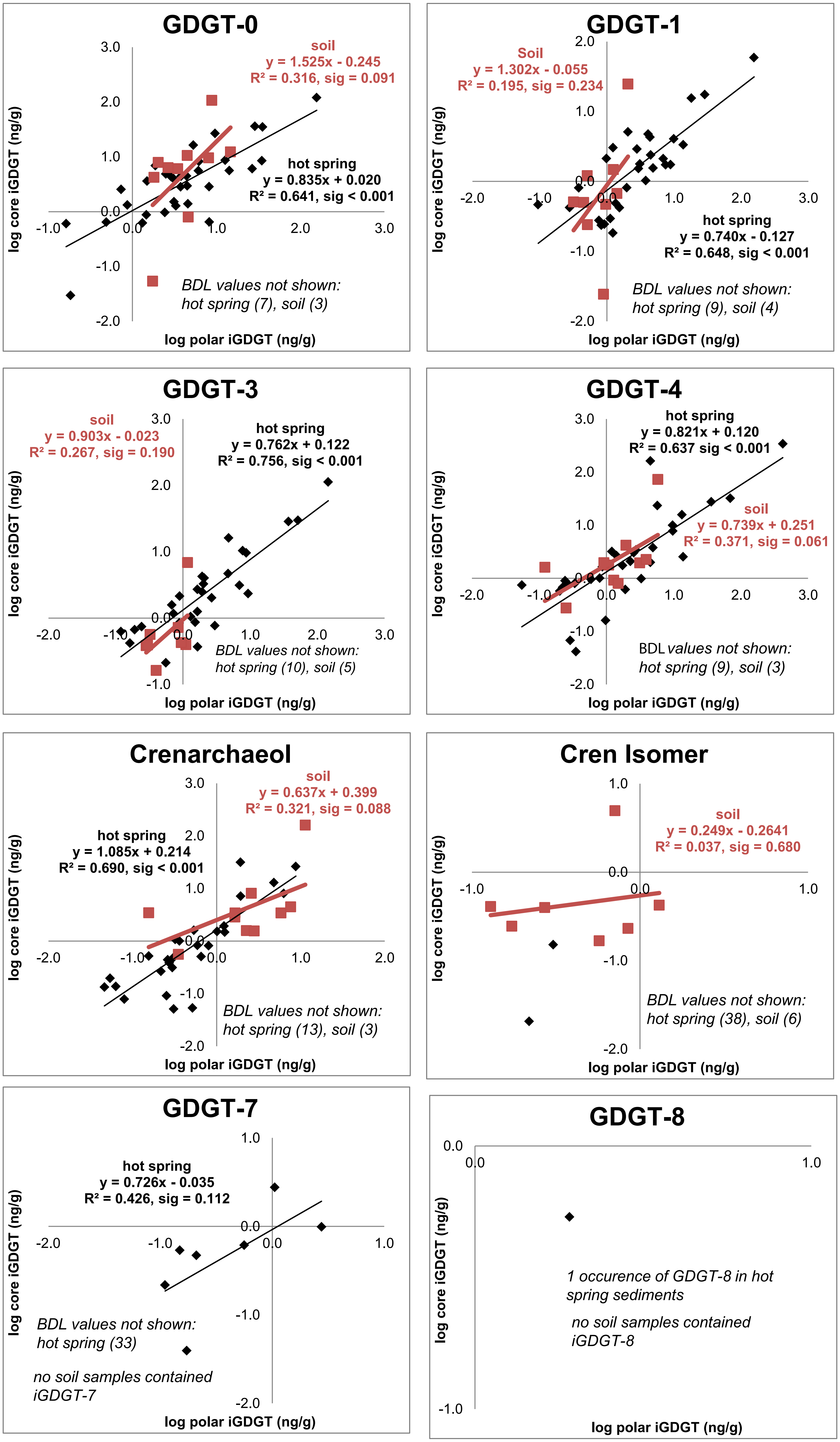

Figure S3: Absolute abundance of log-transformed polar versus core iGDGTs from soil (red) and hot spring (black) samples. Samples below the method detection limit (BDL) for polar iGDGTs are not shown and were not used for in regression analyses (missing values indicated in each plot). Lipid abundance is reported as ng lipid g^{-1} dry mass.
